# Results of extended plant tests using more realistic exposure scenarios for improving environmental risk assessment of veterinary pharmaceuticals

**DOI:** 10.1186/s12302-016-0089-2

**Published:** 2016-08-09

**Authors:** Elisabeth Richter, Silvia Berkner, Ina Ebert, Bernhard Förster, Nadin Graf, Monika Herrchen, Ute Kühnen, Jörg Römbke, Markus Simon

**Affiliations:** 1 ECT Oekotoxikologie GmbH (ECT), 65439 Flörsheim, Germany; 2 German Environment Agency (UBA), 06844 Dessau, Germany; 3 Fraunhofer Institute for Molecular Biology and Applied Ecology (IME), 57392 Schmallenberg, Germany; 4 Institute for Environmental Sciences, University Koblenz-Landau, 76829 Landau, Germany

**Keywords:** Veterinary pharmaceutic products, Antibiotics, Non-extractable residues, Transformation products, Manure, Terrestrial plant test, Phytotoxicity, Environmental risk assessment, Risk refinement

## Abstract

**Background:**

Residues of veterinary medicinal products (VMPs) enter the environment via application of manure onto agricultural areas where in particular antibiotics can cause phytotoxicity. Terrestrial plant tests according to OECD guideline 208 are part of the environmental risk assessment of VMPs. However, this standard approach might not be appropriate for VMPs which form non-extractable residues or transformation products in manure and manure-amended soil. Therefore, a new test design with a more realistic exposure scenario via manure application is needed. This paper presents an extended plant test and its experimental verification with the veterinary antibiotics florfenicol and tylosin tartrate. With each substance, plant tests with four different types of application were conducted: standard tests according to OECD 208 and three tests with application of test substance via spiked manure either without storage, aerobically incubated, or anaerobically incubated for different time periods.

**Results:**

In standard tests, the lowest NOEC was <0.06 mg/kg dry soil for florfenicol and 16.0 mg/kg dry soil for tylosin tartrate. Pre-tests showed that plant growth was not impaired at 22-g fresh manure/kg dry soil, which therefore was used for the final tests. The application of the test substances via freshly spiked as well as via aerobically incubated manure had no significant influence on the test results. Application of florfenicol via anaerobically incubated manure increased the EC10 by a factor up to 282 and 540 for half-maximum and for maximum incubation period, respectively. For tylosin tartrate, this factor amounted to 64 at half-maximum and 61 at maximum incubation period. The reduction of phytotoxicity was generally stronger when using cattle manure than pig manure and particularly in tests with cattle manure phytotoxicity decreased over the incubation period.

**Conclusions:**

The verification of the extended plant test showed that seedling emergence and growth are comparable to a standard OECD 208 test and reliable effect concentrations could be established. As demonstrated in the present study, phytotoxicity of veterinary antibiotics can be significantly reduced by application via incubated manure compared to the standard plant test. Overall, the presented test design proved suitable for inclusion into the plant test strategy for VMPs.

**Electronic supplementary material:**

The online version of this article (doi:10.1186/s12302-016-0089-2) contains supplementary material, which is available to authorized users.

## Background

Residues of veterinary medicinal products (VMPs) enter the environment as unwanted side effect of their extensive use in animal husbandry, either as unchanged active pharmaceutical ingredient (API) or as metabolite (ME) or transformation product (TP). The majority of veterinary pharmaceutical residues enter the environment via application of manure of treated animals onto agricultural areas where they can adversely affect terrestrial plants. Especially, antibiotics are known to cause phytotoxic effects by influencing different plant physiological activities [1].

Terrestrial plant tests are part of the environmental risk assessment (ERA) according to the VICH-guideline Phase II [2] within the marketing authorisation procedure of new VMPs. In the tier A and B risk assessment, the “seedling emergence and growth tests” according to OECD 208 [3] are generally used. The stepwise approach in the risk assessment as well as the particular test requirements is explained in more detail in the reflection paper of the European Medicines Agency (EMA) on testing strategy and risk assessment for plants [4].

However, experience from the regulatory practice has shown that this standard approach might not be appropriate for active substances which form high amounts of non-extractable residues (NER) or TPs in manure and manure-amended soil. The manure matrix consists of a high amount of organic matter and undergoes decomposition during storage and after spreading onto soil. Consequently, NER could be released and the active substance could become bioavailable again. So far, a harmonised concept taking account of NER and TPs in the terrestrial plant testing of VMPs is still lacking [5].

In 2011, the German Environment Agency (Umweltbundesamt) has initiated and funded a 3-year research project on the development of an alternative terrestrial plant test for the risk refinement, especially for antibiotics. With this project, a new test design was developed, applying a more realistic exposure scenario via manure application [6].

The aim of this paper is to illustrate the development of the new testing strategy and the verification of the test design. To this end, florfenicol and tylosin tartrate, two widely used phytotoxic veterinary pharmaceuticals, were assessed in extended plant tests with application via pig and cattle manure and results were compared to those from standard plant tests.

## Materials

### General plant test conditions

All plant tests in this project were conducted according to OECD guideline 208 [3]. The basic experimental procedure is described in the following, while details e.g. the specific test conditions are listed in the Additional file 1.

Tests on florfenicol were conducted at Fraunhofer Institute for Molecular Biology and Applied Ecology (IME), Schmallenberg, Germany, while tests on tylosin tartrate were performed at ECT Oekotoxikologie GmbH, Flörsheim/Main, Germany. If not indicated otherwise, test conditions were identical. As test substrate, the standard soil RefeSol 01-A, a loamy sand, was applied. The soil was sieved at 2 mm before application. The soil was not sterilised. Characteristics of the soil are summarised in Table 1.

**Table 1:** Characteristics of soil RefeSol 01-A used in the plant test

Parameter	Value
Sand (63–2000 µm) (%)	70
Silt (2–63 µm) (%)	27
Clay (<2 µm) (%)	3
C_org_ (%)	1.1
pH (0.01 M CaCl_2_)	5.0
CEC_eff_ (mmolc/kg)	8.6
WHC_max_ (g/kg)	233

Particle size distribution according to E DIN ISO 11277 (2009)

The test substances florfenicol (CAS No. 76639-94-6) and tylosin tartrate (CAS No. 1405-69-0 for tylosin) were purchased from LKT Laboratories, Inc., USA, and Sigma-Aldrich, Germany, respectively. In the standard test, florfenicol was applied via dilutions of an acetonic stock solution spiked on quartz sand due to the low water solubility of florfenicol. After complete evaporation of the solvent, the spiked quartz sand was mixed into the soil (10-g quartz sand/kg soil dry mass). Tylosin tartrate was applied via dilutions of an aqueous stock solution directly to the soil. For the standard control, the same amount of water instead of test solution was applied (for both test substances), while for the solvent control an equal amount of acetone-treated quartz sand was applied (only for florfenicol).

The soil was weighed for each treatment, mixed with water (standard control), equal volumes of test solutions (tylosin tartrate) or spiked quartz sand (florfenicol) to obtain the targeted test substance concentration in the soil. After thorough mixing, the soil was distributed to common plant pots made of polypropylene (florfenicol: 9 cm width, 9 cm length, 12 cm height, 530 g soil fresh mass; tylosin tartrate: diameter 11 cm, height 9 cm, 350 g soil fresh mass). Each pot was placed in a separate polystyrene beaker (e.g. Kastelplast GmbH, Mainz, Germany) serving as water reservoir. At least four replicates (pots) with overall at least 20 seeds were prepared.

The seeds were randomly picked with a pair of tweezers and pressed into the soil at a uniform distance between seeds. The seeds had neither been dressed with fungicides nor imbibed with water. Three monocots and six dicots were chosen for the tests (Table 2) belonging to the crops recommended by guideline OECD 208. Depending on the laboratory and test design, not all mentioned species were tested. Details on plant varieties and origin of seeds are summarised in Additional file 1.

**Table 2:** List of plant species used in plant tests

Species	Family	Common name
*Allium cepa*	Amaryllidaceae	Onion
*Triticum aestivum*	Poaceae	Wheat
*Avena sativa*	Poaceae	Oat
*Solanum lycopersicum*	Solanaceae	Tomato
*Brassica napus*	Brassicaceae	Oilseed rape
*Sinapis alba*	Brassicaceae	Mustard
*Cucumis sativus*	Cucurbitaceae	Cucumber
*Phaseolus vulgaris*	Fabaceae	Common bean
*Trifolium pratense*	Fabaceae	Red clover

Pots were distributed randomly in climate controlled rooms equipped with artificial lighting (at ECT: SON-T-Agro high-pressure metal halide lamps; at IME: SON-T Agro E40, high-pressure sodium lamps; Philips GmbH, Hamburg, Germany; Photo period = 16 h) and targeted conditions of >200 µE/m^2^*s light intensity, 22 ± 10 °C temperature and 70 ± 25 % air humidity. Specific conditions recorded for each test are summarised in the Additional file 1. Pots were covered with transparent lids and water reservoirs were filled with deionised water to moisten the soil sufficiently.

During the test, deionised water was added to the reservoirs as necessary to maintain the soil sufficiently moist. Pots were randomised at minimum weekly intervals, bottom-watered with deionised water as necessary and supplemented with commercially available fertilizer (COMPO, Floragard or Substral Grünpflanzendünger). Nutrients applied via fertilizer corresponded to approximately 30 mg total nitrogen, 15 mg phosphate and 30 mg potassium oxide per kg soil.

The number of seedlings was checked daily until the day on which at least 50 % of the plants in the control (in manure tests the manure control) had emerged. This day was defined as day 0 of the test for each species. Seedlings were also counted on day 7 to determine the emergence rate, and at the end of the study. Survival of seedlings was determined by the difference between the number of seedlings emerged until day 7 and the number of live seedlings at the end of the test. At the end of the test (between day 14 and 21), seedlings were clipped at soil surface, and the shoot length and shoot fresh mass (biomass) of each individual plant was determined immediately. Plants were visually inspected on day 7 or at least at the end of the testing period for any symptoms of impaired health.

Modifications from this standard test procedure that were applied in the extended, modified plant tests with manure are described in the following.

### Handling of manure

**Fig. 1:**
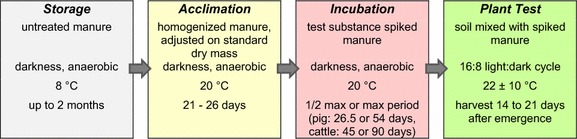
Flow sheet of extended plant tests

The general handling procedure of manure encompassed sampling, storage, processing, acclimation and incubation. A flow sheet is provided in Fig. 1 and basic characteristics of each phase are detailed below.

#### Sampling and storage of manure

The pig and cattle manure originated from animals that were reared under well controlled conditions and the sampling took place at periods where contamination with veterinary medicines and biocides could be excluded. Prior to sampling, the liquid manure in the tank was stirred for 1 h. Liquid manure was collected from the tank by a ladle with a large beaker, and filled into 60 L barrels. The barrels were sealed and stored at 8 °C in the dark under anaerobic conditions. Expanding gas was allowed to escape once or twice a week. Storage duration for the pre-tests was 3–4, 10 weeks for the range-finding tests and 3 weeks for the main tests.

#### Processing and acclimation

To obtain standardised testing conditions, the dry matter content in the pig and cattle manure was adjusted to 5 ± 1 % and 10 ± 1 %, respectively, according to recommendations by EMA [7]. The dry matter content of manure was chosen as reference parameter as it is expected to be more relevant than the nitrogen concentration on the effects of veterinary medicinal products in plant tests. Dry matter content of the original manure was determined (5.0 % in pre-tests and 11.8 % in main tests for pig manure, 10.0 % in pre-tests and 9.8 % in main tests for cattle manure) and lowered when necessary by adding the respective volume of water (deionised water, bubbled with nitrogen for 30 min).

Manure was then shredded using a hand mixer in order to obtain a homogenous and fairly stable phase and dry matter content of the processed manure was determined. To prevent introduction of oxygen, mixing was conducted in a sealed beaker flooded with nitrogen. Subsequently, the processed manure was filled into acclimation containers (20 L buckets) up to approximately 75–80 % of maximum container volume. After flooding the container with nitrogen to ensure anaerobic conditions, it was sealed but allowed gas to escape.

Then, an acclimation phase followed where the processed manure was stored at 20 °C in the dark for 21–26 days.

#### Spiking and incubation of manure

Manure was added at 22-g fresh mass/kg soil dry mass, corresponding to approximately 85 kg N/ha, the half-maximum permitted annual application rate in Europe [8]. This concentration had proven to cause no detrimental effects on seedling emergence and growth in the pre-tests (see “Effects of pig and cattle manure application on seedling emergence and growth (pre-tests**)**” section). The test concentrations and experimental procedure were always the same for pig and cattle manure.

Details on test concentrations and replication for each test and plant species are provided in the supplementary information (Additional file 1: Tables S3, S4).

Florfenicol was applied via dilutions of an acetonic stock solution. Test solutions (fixed volumes within one test, <600 µL) were spiked to a portion of manure (22 g fresh mass/kg soil dry mass) and mixed in 150-mL glass beakers. Evaporation of the solvent was accelerated by lowering air pressure in an anaerobic cabinet three times to approximately 500 mbar for 1 min. To investigate effects of anaerobic incubation, the incubation was conducted in an anaerobic cabinet under anaerobic conditions at 20 ± 2 °C in the dark. To investigate effects of aerobic incubation, 44-g fresh mass of freshly spiked manure was mixed into 1.6 kg of soil. To increase homogeneity of distribution, the manure was first mixed with a subsample of the soil before adding the remaining soil. This substrate was placed into containers of nonporous plastic, adjusted to 40 % of maximum water holding capacity and incubated under aerobic conditions at 20 ± 2 °C in the dark for half-maximum storage duration.

For tylosin tartrate, the respective amount of test substance was directly weighed into 50-mL glass beakers and thoroughly mixed with a portion of manure (22-g fresh mass/kg soil dry mass, mixed with a subsample of soil, thoroughly homogenised and then mixed with the rest of the soil batch).

#### Application of spiked manure in the plant test

To simulate a more realistic exposure route of veterinary antibiotics, the test substances were applied via spiked manure to the soil of the plant tests. Three scenarios with both pig and cattle manure were tested for the application via manure: (1) application of freshly spiked manure, (2) application after half-maximum anaerobic incubation of manure, and (3) application after maximum anaerobically incubation of manure. Additionally, freshly spiked manure was mixed into soil and incubated aerobically for the half-maximum storage duration (ageing) before starting the plant test.

The incubation time was derived from the default values for manure storage time summarised in EMEA/CVMP/ERA/418282 [9]. In our tests, maximum and half-maximum incubation periods corresponded to 53 and 26.5 days, respectively, for pig manure, and 90 and 45 days, respectively, for cattle manure.

For anaerobic incubation, the spiked manure was incubated for the respective incubation time at 20 ± 2 °C in the dark. The incubation took place in nitrogen-flooded screw cap bottles of amber glass (tylosin tartrate) or in open glass vessels in a nitrogen-flooded anaerobic chamber (florfenicol). After the particular incubation period, the manure was added to a subsample of soil, thoroughly mixed, and then homogenised with the residual soil mass.

Effects of aerobic incubation were tested for the half-maximum incubation period. For this purpose, freshly spiked manure was added to soil (first at a ratio of 88 g fresh mass manure to 800 g dry mass soil, then mixed thoroughly with the residual 3200 g soil dry mass). The mixture was adjusted to 40 % of maximum water holding capacity, placed into containers of nonporous plastic and incubated in the dark under aerobic conditions at 20 ± 2 °C for 26.5 days (pig manure) and 45 days (cattle manure).

#### Chemical analysis of florfenicol and tylosin tartrate

^14^C-Florfenicol extracted from manure was quantified by liquid scintillation counting (LSC) and analysed for the test item by TLC-analysis. The extracted manure was analysed for non-extractable residues by combustion with subsequent liquid scintillation counting (LSC) of the formed ^14^CO_2_. Unlabelled florfenicol and tylosin tartrate were quantified by high performance liquid chromatography (HPLC). A full description of the analytical methods is given in [2].

#### Monitoring of key parameters during manure processing

Key parameters of the manure as well as redox potential and pH were measured during sampling, at the end of the storage and at the end of the acclimation phase. The applied methods are summarised in Table 3.

**Table 3:** Parameters measured in manure at different stages and methods applied

Parameter	Method	Determined during sampling (1), end of storage (2), end of acclimation (3)
pH	Direct measurement in liquid manure	1, 2, 3
Temperature	Direct measurement in liquid manure	1, 2, 3
Redox potential	Direct measurement in liquid manure	1, 2, 3
Dry matter content	Drying of subsamples of 10-g fresh mass at 105 °C overnight in a drying chamber. Quantification by weighting	2, 3
Microbial activity	For testing the microbial activity several suggestions exist. In this case it was mineralisation of ^14^C-labeled glucose under anaerobic conditions	3
NH_4_-N content	Ammonium-N, purge and trap-method. Quantification by titration [10]	3
Nonvolatile N content	Analysis of dried manure by determination with element analyser after combustion	3
Total N content	Calculation by addition of NH_4_–N content and nonvolatile N content	3
Organic C content	Analysis of dried manure by determination with element analyser after combustion	3
Total P content	Extraction of trace elements soluble in aqua regia [11] and phosphor quantification by ICP-OES with a matrix adjusted calibration [12]	3
Total Cu content	Extraction of trace elements soluble in aqua regia [10] and phosphor quantification by ICP-OES with a matrix adjusted calibration [12]. Copper quantification by ICP-OES with a matrix adjusted calibration	3

### Pre-test on effects of manure application rates on seedling emergence and growth

Pre-tests were conducted in order to determine up to which manure concentrations seedling emergence and growth would not be impaired. Five manure concentrations were selected based on the European maximum application rate of 170 kg N/ha to be deployed in 1 year [8]: 7, 11, 18, 29 g/kg and 46 g manure fresh mass/kg soil dry mass, corresponding to approximately 26, 42, 66, 106 and 170 kg N/ha (based on the average content of total nitrogen in the manure of 4.91 g total N/kg manure fresh mass and assuming an incorporation depth of 0–5 cm and a soil density of 1.5 g/cm^3^).

The manure was sampled, stored, processed and acclimated as described above.

Eight plant species were tested: *Allium cepa*, *Brassica napus*, *Sinapis alba*, *Solanum lycopersicum,Phaseolus vulgaris*, *Triticum aestivum, Cucumis sativus* and *Trifolium pratense*.

### Standard plant test on florfenicol and tylosin tartrate

General phytotoxicity of the two antibiotics florfenicol and tylosin tartrate was determined in standard plant tests (i.e. without manure) conducted under the previously described test conditions.

For florfenicol, five treatments with nominal test concentrations between 0.06 mg/kg soil dry mass and 5.0 mg/kg soil dry mass were tested. There were four or five replicates with five seeds (*A. cepa*, *Avena sativa*, *B. napus*, *S. alba* and *S. lycopersicum*) and four seeds (*P. vulgaris*), respectively, to give a total of 20 seeds per treatment and control.

As florfenicol is not sufficiently water soluble, acetone was used as solvent to prepare the treatment solutions. Quartz sand (10 g/kg soil dry mass) was used as carrier and spiked with the treatment solutions. After evaporation of acetone, the spiked quartz sand and the respective amount of water needed to adjust the soil to 40 % of maximum water holding capacity were added to the test soil and mixed thoroughly.

Based on previous tests demonstrating that acetone evaporated overnight completely and no adverse effect occurred, only the solvent control (i.e. no additional standard control) was applied.

For tylosin tartrate, five treatments per species with species-specific ranges between 9.3 and 800 mg/kg soil dry mass were tested against an untreated standard control.

As tylosin tartrate is sufficiently water soluble, test solutions were obtained by diluting defined volumes of stock solutions with deionised water. A defined volume of the appropriate test solution was mixed into the bulk soil for the respective treatment. The untreated control soil was mixed with the same volume of deionised water.

After mixing the test solution into the bulk soil for each treatment, the soil was distributed to the pots and seeds were sown. There were four replicates with three seeds (*P. vulgaris*) or five replicates with four seeds (*A. cepa*, *A. sativa*, *B. napus*, *S. alba*, *S. lycopersicum, T. pratense*), respectively, to give a total of 12 (*P. vulgaris*) or 20 seeds (other species) per treatment and control.

### Extended plant tests with test substances applied via spiked manure

For florfenicol, the number of replicates (pots) per treatment and species was eight for treatments with manure and five or four for the standard control. The number of seeds per pot was five (*A. cepa*, *A. sativa*, *B. napus*, *S. lycopersicum, S. alba*), or four (*P. vulgaris*) so that the total number of seeds per species was 40 or 32 for treatments and manure control and 20 for the standard control.

For tylosin tartrate, the number of replicates (pots) per treatment and species was eight for treatments with manure and five or four for the standard control. The number of seeds per pot was five (*A. cepa*, *A. sativa*, *B. napus*, *S. lycopersicum, T. pratense*), or three (*P. vulgaris*) so that the total number of seeds per species was 40 or 24 for treatments and manure control and 20 or 12 for the standard control.

For the aerobic half-maximum incubation, only the two most sensitive species, i.e. *A. cepa* and *B. napus* (florfenicol) or *A. cepa* and *T. pratense* (tylosin tartrate), were tested.

Nominal test concentrations of florfenicol in tests with freshly spiked manure ranged from 0.20 to 16.7 mg/kg soil dry mass, tested against a manure control (acetone spiked manure added to soil) and a standard control (only soil). In tests with half-maximum and maximum incubation period, florfenicol concentrations ranged from 0.62 to 50.0 mg/kg soil dry mass.

Nominal test concentrations of tylosin tartrate in all extended plant tests ranged from 5.0 to 1000 mg/kg soil dry mass, tested against a manure control (manure added to soil) and a standard control (only soil).

### Data evaluation and statistics

For all tests, validity criteria according to OECD 208 [3] were checked. Biological results were evaluated as  % inhibition compared to the standard control (in standard tests) or the manure control (in extended tests).

Effect values were calculated based on nominal initial concentrations referring to soil dry mass.

Probit analysis was used to determine effect concentrations EC_10_ and EC_50_. Seedling emergence and survival data were checked by the Fisher’s exact binominal test with Bonferroni correction to determine the no observed effect concentration NOEC at a significance level of *α* = 0.05, one-sided greater.

For shoot length and biomass data, normal distribution was checked by Shapiro–Wilk’s Test, and homogeneity of variances by Levene’s Test. The NOEC was determined applying the Williams Test for homogeneous variances, and the Welch *t* Test for inhomogeneous variances, at a significance level of *α* = 0.05, one-sided smaller.

Effect concentrations and NOECs were performed using the ToxRat software Version 2.10 (ToxRat Solutions GmbH, 2010).

Effect concentrations (EC_10_ or EC_50_) of extended plant tests on florfenicol were evaluated for the influence of incubation duration (no incubation/half-max/max) and the type of manure (pig/cattle). Normal distribution of errors and variance homogeneity were checked via visual inspection and Levene’s test. As data were heteroscedastic, the non-parametric Duncan Test (*a* = 0.05) was performed using the program STATISTICA (version 10). For tylosin tartrate, this evaluation could not be performed as many effect concentrations could not be determined.

## Results

### Effects of pig and cattle manure application on seedling emergence and growth (pre-tests)

The most sensitive endpoint was seedling emergence, which is summarised for the tested six (pig manure) and eight (cattle manure) plant species, respectively, in Table 4.

**Table 4:** Seedling emergence (% of seeds sown) in pre-tests with manure (cattle/pig)

Manure concentration (kg N/ha)	*A. cepa*	*T. aestivum*	*P. vulgaris*	*T. pratense*	*B. napus*	*S. lycopersicum*	*C. sativus* ^a^	*S. alba* ^a^
0	75/75	100/71	80/80	88/88	88/96	72/88	83.3	76
26.0	96/83	100/54	70/90	92/71	96/75	85/97	95	97
42.0	88/92	100/67	95/90	86/79	97/80	85/98	99	89
66.0	71/96	100/50	90/100	92/79	98/85	90/98	96	95
106	79/83	100/63	65/75	79/58	94/65	90/96	91	93
170	79/92	100/54	60/65	71/67	97/70	85/89	89	88

^a^ *C. sativus* and *S. alba* were only tested with pig manure

In the standard controls, seedling emergence of at least 71 % of seeds sown (*T. aestivum*) was observed. To meet the validity criteria of the test guideline OECD 208, the seedling emergence rate must exceed 70 % in the controls. Seedling emergence of less than 70 % was observed at cattle manure application rates of 106 kg N/ha (29 g manure/kg soil dry mass) in *P. vulgaris*. In tests with pig manure, seedling emergence of less than 70 % occurred in *T. aestivum* at ≥26 kg N/ha, in *B. napus* and *T. pratense* at ≥106 kg N/ha, and in *P. vulgaris* at 170 kg N/ha. Only *A. cepa* was not affected by pig manure rates up to and including 170 kg N/ha.

A statistically significant impairment of the seedling emergence rate compared to that in the controls (Fisher’s Exact Binomial Test with Bonferroni Correction: pair-wise comparisons between treatment and control on the multiple significance level with *α* = 0.05, one-sided greater) was detected only for pig manure at 106 kg N/ha in *B. napus* and at 170 kg N/ha in *S. lycopersicum* and *C. sativus*.

Post-emergence survival was affected only in *B. napus* (21 % mortality) at the highest pig manure application rate of 170 kg N/ha (for details see Additional file 1). Shoot length and biomass were not impaired by pig manure up to and including 170 kg N/ha, only in *S. alba*, it was reduced by 34 % at the highest tested rate. Cattle manure had no negative effects on plant growth in any of the species up to 170 kg N/ha.

To avoid any plant growth impairment from the manure amendment, a concentration of 22 g fresh mass manure/kg soil dry mass, corresponding to approximately 85 kg N/ha, was chosen for the final tests.

In the pre-tests, *T. aestivum* and *C. sativus* had shown generally moderate emergence and/or survival rates; therefore, these species were not applied in further tests. Instead, *A. sativa* was tested as second monocot species (replacing *T. aestivum*). Due to impaired seedling emergence at either laboratory test site, *T. pratense* was not used in tests with florfenicol (conducted at IME) while *S. alba* was not applied in tests with tylosin tartrate (conducted at ECT).

### Characteristics of manure applied in plant tests

Pig and cattle manure applied in pre-tests differed particularly in their organic matter content (5 and 10 %, respectively) while pH and nitrogen contents were similar (Table 5).

**Table 5:** Characteristics of manure applied in modified plant tests with florfenicol and tylosin tartrate

Parameter	Pig manure	Cattle manure
*Manure characterisation on sampling site*		
Temperature (°C)	18 (12.5)^a^	11 (5.8)^a^
pH	7.7 (7.8)	6.9 (7.6)
Redox potential (mV)	−410 (−374)	−345 (−362)
*Manure characterisation after storage*		
Temperature (°C)	8.0	8.0
pH	7.6	6.9
Redox potential (mV)	−388	−295
Dry matter content (originally) (%)	11.8 (5.0)	9.8 (10.0)
Dry matter content (after processing) (%)	4.8 (5.0)	9.6 (10.0)
*Manure characterisation after acclimation*		
Temperature (°C)	20	20
pH	7.4	6.8
Redox potential (mV)	−367	−327
Dry matter content (%)	4.8	9.6
NH_4_–N (mg/kg fresh weight)	1740	1500
Nonvolatile nitrogen (mg/kg fresh weight)	1258	2102
Total nitrogen (calculated) (mg/kg fresh weight)	2998	3602
Organic carbon (mg/kg fresh weight)	17,380	39,725
Total P content (mg/kg fresh weight)	1320	820
P2O5 equivalents (mg/kg fresh weight)	3024	1877
Total Cu content (mg/kg fresh weight)	14	20
Microbial activity (in 7 days)	36 % glucose degradation (32 % CO_2_, 4 % CH_4_)	59 % glucose degradation (50 % CO_2_, 9 % CH_4_)

^a^Values in brackets refer to manure used in the pre-tests

In the extended plant tests, manure was prepared to reach approximately 5 % (pig, measure: 4.8 %) and 10 % (cattle, measure: 9.6 %) dry matter, respectively (Table 5). Correspondingly, the organic carbon content was substantially greater in cattle than in pig manure. The pH was slightly higher in pig compared to cattle manure (7.7 and 6.9, respectively) during sampling and appeared to slightly decrease with incubation (7.4 and 6.8, respectively). Anaerobic conditions remained stable during the incubation as demonstrated by redox potential values (Eh < −100 mV; Table 5).

### Analytical verification of test substance concentrations at test start

For florfenicol, chemical analysis of the highest application solutions showed generally good recovery, with 85–110 % for all tests (no analysis in standard test) (see Table 6). Analysis via radio-labelled substance of extractable residues in manure spiked at 250 mg/kg soil fresh mass indicated that florfenicol’s bioavailability seems to be reduced by about 40 % at half-maximum and by about 60 % at maximum incubation time.

**Table 6:** Measured concentrations of the test substances in highest application solution (florfenicol) and in spiked manure (tylosin tartrate), respectively, after anaerobic incubation for different periods of time

Substance	Day of incubation	Florfenicol^a^ recovery in highest application solution (%)	Tylosin tartrate^b^ recovery in spiked manure at representative test concentrations (%)
Standard test	–	n.d.	n.d.
Fresh spiked pig manure	0	85–95	6.0–38.2
Fresh spiked cattle manure	0	94–110	6.5–34.9
Half-max incubated pig manure	27	94–100	8.1–35.2
Half-max incubated cattle manure	45	103–104	16.7–27.1
Max incubated pig manure	53	94–100	11.4–31.9
Max incubated cattle manure	90	103–104	14.9–30.4

^a^Florfenicol was applied via an acetonic application solution

^b^Tylosin tartrate was applied via dry mixing into dung and analysed in pig manure at 0.228/1.365/4.0/7.1/17.8/45.5 mg/kg soil dry mass, in cattle manure at 0.228/1.365/4.0/7.1/17.8/45.5 mg/kg soil dry mass (fresh and maximum incubated manure) and at 0.228/1.365 (half-maximum incubated manure)

For tylosin tartrate, which had been directly weighed into manure, recovery in spiked manure was generally rather poor, both in freshly spiked as well as incubated manure (6.0–35.2 %, no analysis in standard test). Even when directly analysing freshly spiked manure, only 63 % recovery was obtained. Overall, the application via a test solution and its analytical verification is recommended by the OECD 208 test guideline. However, it can be assumed that little loss occurred by directly weighing and spiking of tylosin tartrate. Therefore, data evaluation and statistical analyses were generally based on nominal initial concentrations.

### Toxicity of florfenicol and tylosin tartrate assessed in standard plant tests

Florfenicol application caused no pathological symptoms besides a light chlorosis in *S. alba*, *B. napus* and *S. lycopersicum* seedlings.

Emergence of *S. lycopersicum, S. alba* and *P. vulgaris* was clearly affected by florfenicol with the lowest NOEC of <0.06 mg/kg soil dry mass for *S. lycopersicum*. The lowest EC_10_ with 0.01 mg/kg soil dry mass was calculated for *B. napus*, the lowest EC_50_ with 1.46 mg/kg soil dry mass for *S. alba*. No or little impairment of seedling emergence was observed in *A. cepa*, *A. sativa*, and *B. napus* up to and including 5.0 mg/kg soil dry mass, the highest concentration tested.

Post-emergence survival of *A. cepa*, *B. napus*, *S. alba*, and *S. lycopersicum* was clearly affected by florfenicol. The most sensitive species regarding post-emergence survival was *S. alba* with a NOEC of 0.56 mg/kg soil dry mass, an EC_10_ of 0.24 mg/kg soil dry mass, and an EC_50_ of 0.60 mg/kg soil dry mass. There was no or only a slight concentration dependent effect of florfenicol on post-emergence survival of *A. sativa* and *P. vulgaris* up to and including 5.0 mg/kg soil dry mass.

Growth in terms of biomass (shoot fresh mass) and shoot length was affected by florfenicol in a concentration dependent manner in all species tested. The most sensitive species regarding shoot length and biomass was *B. napus* with a NOEC of 0.06 mg/kg soil dry mass and <0.06 mg/kg soil dry mass, respectively.

In summary, florfenicol negatively affected emergence and growth of all six tested plant species with biomass (fresh mass) being generally the most sensitive endpoint (Table 7). The lowest EC_10_ (0.05 mg/kg soil dry mass) and EC_50_ (0.25 mg/kg soil dry mass) were found for biomass of *B. napus*, with *A. cepa* and *S. alba* being similarly sensitive. The lowest NOEC (<0.06 mg/kg soil dry mass) was observed for emergence of *S. lycopersicum* and biomass of *B. napus*.

**Table 7:** Toxicity of florfenicol (mg/kg soil dry mass) toward six plant species assessed in standard plant tests

Species	*A. cepa*	*A. sativa*	*B. napus*	*S. alba*	*S. lycopersicum*	*P. vulgaris*
*Emergence*						
NOEC	≥5.00	≥5.00	≥5.00	0.56	<0.06	1.67
EC_10_	6.81	0.34	0.01	0.12	n.d.	0.57
	(n.d.)	(0.0–0.12)	(n.d.)	(0.03–0.25)	(–)	(0.13–1.13)
EC_50_	n.d.	>5.00	>5.00	1.46	>5.00	>5.00
	(–)	(–)	(–)	(0.84–3.11)	(–)	(–)
*Post-emergence survival*						
NOEC	1.67	≥5.00	1.67	0.56	0.56	≥5.00
EC_10_	0.35	2.28	0.71	0.24	0.43	n.d.
	(0.06–0.75)	(0.04–303)	(n.d.)	(0.09–0.36)	(0.09–0.85)	(–)
EC_50_	4.82	>5.00	2.71	0.6	3.43	n.d.
	(2.13–35.8)	(–)	(n.d.)	(0.40–1.00)	(1.75–14.9)	(–)
*Shoot length*						
NOEC	0.19	≥5.00	0.06	0.19	0.56	0.56
EC_10_	0.16	>5.00	0.06	0.14	0.25	0.74
	(0.14–0.19)	(–)	(0.0–0.17)	(n.d.)	(n.d.)	(0.42–1.03)
EC_50_	2.57	n.d.	0.93	0.60	1.83	2.86
	(2.36–2.80)	(–)	(0.46–2.11)	(n.d.)	(n.d.)	(2.35–3.53)
*Biomass*						
NOEC	0.06	1.67	<0.06	0.19	0.56	0.56
EC_10_	0.06	0.58	0.05	0.07	0.46	0.76
	(0.04–0.09)	(n.d.)	(0.01–0.09)	(n.d.)	(n.d.)	(0.36–1.12)
EC_50_	0.75	>5.00	0.25	0.32	0.76	2.59
	(0.63–0.89)	(–)	(0.16–0.38)	(n.d.)	(n.d.)	(2.02–3.36)
Valid	Yes	Yes	Yes	Yes	Yes	Yes

Effect concentrations (EC_10_, EC_50_ with 95 % confidence interval) and NOEC based on nominal test concentrations with 95 % confidence intervals (CI)

Florfenicol application range (all species): 0.06–5.0 mg/kg

*n.d.* not determined due to inappropriate data

Tylosin tartrate application caused no pathological symptom besides a light chlorosis in seedlings of all species.

Tylosin tartrate had no adverse effect on seedling emergence at concentrations up to and including 83.3 mg/kg soil dry mass (*T. pratense*), 144 mg/kg soil dry mass (*B. napus, S. lycopersicum*), 250 mg/kg soil dry mass (*A. cepa*) and 800 mg/kg soil dry mass (*A. sativa* and *P. vulgaris*) (Table 8).

**Table 8:** Toxicity of tylosin tartrate (mg/kg soil dry mass) toward six plant species assessed in standard plant tests

Species	*A. cepa*	*A. sativa*	*B. napus*	*S. lycopersicum*	*P. vulgaris*	*T. pratense*
*Emergence*						
NOEC	≥250	≥800	≥144	≥144	≥800	≥83.3
EC_10_	n.d.	n.d.	n.d.	n.d.	n.d.	n.d.
	(–)	(–)	(–)	(–)	(–)	(–)
EC_50_	n.d.	n.d.	n.d.	n.d.	n.d.	n.d.
	(–)	(–)	(–)	(–)	(–)	(–)
*Post-emergence survival*						
NOEC	83.3	≥800	83.3	≥144	≥800	48.1
EC_10_	71.2	n.d.	n.d.	104	n.d.	28.1
	(n.d.)	(–)	(–)	(n.d.)	(–)	(n.d.)
EC_50_	135	n.d.	82.6	n.d.	n.d.	57.6
	(n.d.)	(–)	(n.d.)	(–)	(–)	(n.d.)
*Shoot length*						
NOEC	27.8	200	48.1	72.2	100	16.0
EC_10_	14.5^a^	565	54.9	63.2	32.8^a^	11.7
	(0.1–32.3)	(n.d.)	(53.7–55.9)	(45.7–74.8)	(1.0–82.0)	(5.5–17.2)
EC_50_	82.9	n.d.	96.0	127	434	49.8
	(45.8–344)	(–)	(94.9–97.2)	(115–147)	(253–1341)	(39.9–66.4)
*Biomass*						
NOEC	27.8	50	48.1	≥144	≥800	16.0
EC_10_	10.9^a^	113	35.2	45.4	9.1^a^	7.7
	(0.1–22.6)	(14.5–203)	(34.8–35.5)	(40.5–49.5)	(3.0–17.4)	(0.1–15.3)
EC_50_	41.3	603	61.9	74.0	107	23.5
	(15.8–63.3)	(405–1394)	(61.7–62.2)	(70.5–77.6)	(79.4–137)	(3.6–103)
Valid:	Yes	Yes	No (60 % emerged)	No (60 % emerged)	No (58 % emerged)	Yes
Application range	27.8–250	50.0–800	16.0–144	36.1–144	50.0–800	9.30–83.3

Effective concentrations (EC_10_, EC_50_ with 95 % confidence interval) and NOEC based on nominal test concentrations with 95 % confidence intervals (CI)

*n.d.* not determined due to inappropriate data

^a^Extrapolated value (beyond tested concentration range)

The lowest observed concentration to increase seedling mortality was 83.3 mg/kg soil dry mass (*T. pratense*) and 144 mg/kg soil dry mass (*A. cepa* and *B. napus*), respectively.

Tylosin tartrate exhibited clear concentration depending effects on the growth of all species with biomass of *T. pratense* being most sensitive.

In summary, tylosin tartrate negatively affected emergence and growth of all six tested plant species with biomass (fresh mass) being generally the most sensitive endpoint. The lowest EC_10_ (7.7 mg/kg soil dry mass) and EC_50_ (23.5 mg/kg soil dry mass) were found for biomass of *T. pratense*, with *A. cepa* and *P. vulgaris* being only slightly less sensitive. The lowest NOEC (16.0 mg/kg soil dry mass) was observed for shoot length and biomass of *T. pratense*.

### Toxicity of florfenicol and tylosin tartrate assessed in extended plant tests

There was no noticeable adverse effect of pig and cattle manure at the applied concentration on seedling emergence, survival and growth in any of the species.

The application of the two antibiotics via manure generally reduced the negative effects on plants. In the following, results are presented and discussed for the biomass of *B. napus* and *P. vulgaris* as representative species being very sensitive to florfenicol (Table 9) and tylosin tartrate (Table 10); more data are provided in the Additional file 1.

**Table 9:** Effect concentrations (EC_10_, EC_50_ with 95 % confidence interval) and NOEC, (mg/kg soil dry mass) for biomass production of *B. napus* and *P. vulgaris* of florfenicol spiked via differently treated manure

Manure	Species/endpoint	Standard test (no manure)	Fresh spiked	1/2 max incubation time	Max. incubation time
	*B. napus*				
Cattle	NOEC	<0.06	<0.2	5.60	16.7
	EC_10_	0.05	0.07	12.1	27.0
		(0.01–0.09)	(n.d.)	(n.d.)	(n.d.)
	*F*		*1.4*	*242*	*540*
	EC_50_	0.25	0.2	20.8	68.0
		(0.16–0.38)	(n.d.)	(n.d.)	(n.d.)
	*F*		*0.8*	*83*	*272*
Valid pig		Yes	Yes	Yes	Yes
	NOEC	<0.06	<0.2	16.7	≥16.7
	EC_10_	0.05	0.08	14.1*	14.3
		(0.01–0.09)	(0.07–0.09)	(14.0–14.2)	(n.d.)
	*F*		*1.6*	*282*	*286*
	EC_50_	0.25	0.21	22.5*	27.8
		(0.16–0.38)	(0.21–0.22)	(22.3–22.7)	(n.d.)
	*Factor*		*0.84*	*90*	*111*
Valid		Yes	No (67 % emerged, 60 % survived)	Yes	No (80 % emerged)
	*P. vulgaris*				
Cattle	NOEC	0.56	<0.2	5.60	5.60
	EC_10_	0.76	0.08	11.8*	8.14*
		(0.36–1.12)	(0.03–0.16)	(9.14–14.2)	(5.86–10.3)
	*F*		*0.1*	*16*	*11*
	EC_50_	2.59	1.18	28.7*	69.7*
		(2.02–3.36)	(0.81–1.67)	(25.6–32.3)	(58.3–88.4)
	*F*		*0.5*	*11*	*27*
Valid pig		Yes	Yes	Yes	Yes
	NOEC	0.56	<0.2	16.7	16.7
	EC_10_	0.76	0.07	11.8	28.2*
		(0.36–1.12)	(0.00–0.27)	(n.d.)	(24.2–32.2)
	*F*		*0.1*	*16*	*37*
	EC_50_	2.59	1.0	27.0	41.2*
		(2.02–3.36)	(0.26–2.77)	(n.d.)	(39.1–42.6)
	*F*		*0.4*	*10*	*16*
Valid		Yes	No (80 % emerged)	Yes	Yes

Concentration range (both species): 0.06–5.0 mg/kg soil (standard tests), 0.2–16.7 mg/kg soil (fresh spiked), 0.62–50.0 mg/kg soil (other tests). Results evaluated as  % inhibition compared to the standard control (in standard tests) or the manure control (in extended tests)

*F* factor of difference*, ECx* (manure spiked test)/*ECx* (standard test)

* Significantly greater than value from standard test (based on overlapping 95 % confidence intervals)

**Table 10:** Effect concentrations (EC_10_, EC_50_ with 95 % confidence interval) and NOEC, in mg/kg soil dry mass) for biomass production of *B. napus* and *P. vulgaris* of tylosin tartrate spiked via differently treated manure

Manure	Species/endpoint	Standard test (no manure)	Fresh spiked	1/2 max incubation time	Max. incubation time
	*B. napus*				
Cattle	NOEC	48.1	25	156	156
	EC_10_	35.2	36.7	254	78.7
		(34.8–35.5)	(25.2–44.9)	(n.d.)	(n.d.)
	*F*		*1.0*	*7.2*	*2.2*
	EC_50_	61.9	78.7*	326	351
		(61.7–62.2)	(69.2–90.3)	(n.d.)	(n.d.)
	*F*		*1.3*	*5.3*	*5.7*
	Valid	No (60 % emerged)	Yes	Yes	Yes
Pig	NOEC	48.1	62.5	156	≥391
	EC_10_	35.2	101	n.d.	n.d.
		(34.8–35.5)	(n.d.)	(–)	(–)
	*F*		*3*	*n.d.*	*n.d.*
	EC_50_	61.9	131	n.d.	n.d.
		(61.7–62.2)	(n.d.)	(–)	(–)
	*F*		*2.1*	*n.d.*	*n.d.*
	valid	No (60 % emerged)	Yes	Yes	Yes
	*P. vulgaris*				
Cattle	NOEC	≥800	40	194	194
	EC_10_	9.1	25.3	175	202*
		(3.0–17.4)	(1.1–59.9)	(n.d.)	(33.6–315)
	*F*		*2.8*	*19*	*22*
	EC_50_	107	177	370	525*
		(79.4–137)	(88–346)	(n.d.)	(352–819)
	*F*		*1.7*	*3.5*	*4.9*
	valid	No (58 % emerged)	Yes	Yes	Yes
Pig	NOEC	≥800	40	426	426
	EC_10_	9.1	18.1	581	553
		(3.0–17.4)	(1.1–43.6)	(n.d.)	(n.d.)
	*F*		*2.0*	*64*	*61*
	EC_50_	107	142	837	885
		(79.4–137)	(72.5–253)	(n.d.)	(n.d.)
	*F*		*1.3*	*8*	*8*
	Valid	No (58 % emerged)	Yes	Yes	Yes

Concentration range (*B. napus*): 16.0–144 and 50–800 mg/kg soil (standard test), 15.0–300 mg/kg soil (fresh spiked), 5.0–391 mg/kg soil (other tests); concentration range (*P. vulgaris*): 50–800 mg/kg soil (standard test), 40–937 mg/kg soil (other tests). Results evaluated as  % inhibition compared to the standard control (in standard tests) or the manure control (in extended tests)

*F* factor of difference*, ECx* (manure spiked test)/*ECx* (standard test)

* Significantly greater than value from standard test (based on overlapping 95 % confidence intervals)

**Fig. 2:**
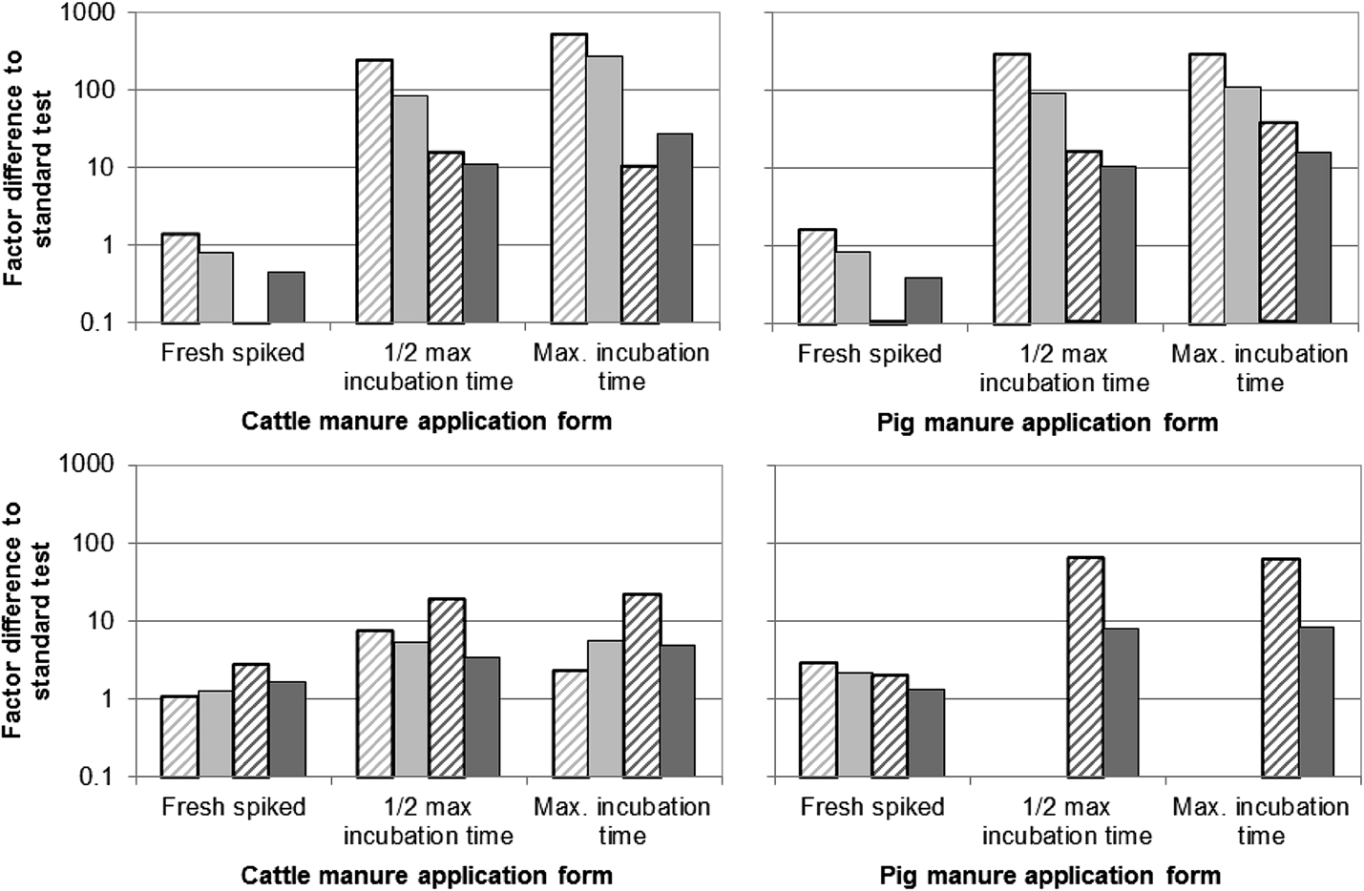
Factor difference between EC_*x*_ from tests with spiked manure to those from standard tests. *Top* florfenicol, *bottom* tylosin tartrate. *Striped bars* EC_10_, *solid bars* EC_50_, *bright grey* *B. napus*, *dark grey* *P. vulgaris*

**Fig. 3:**
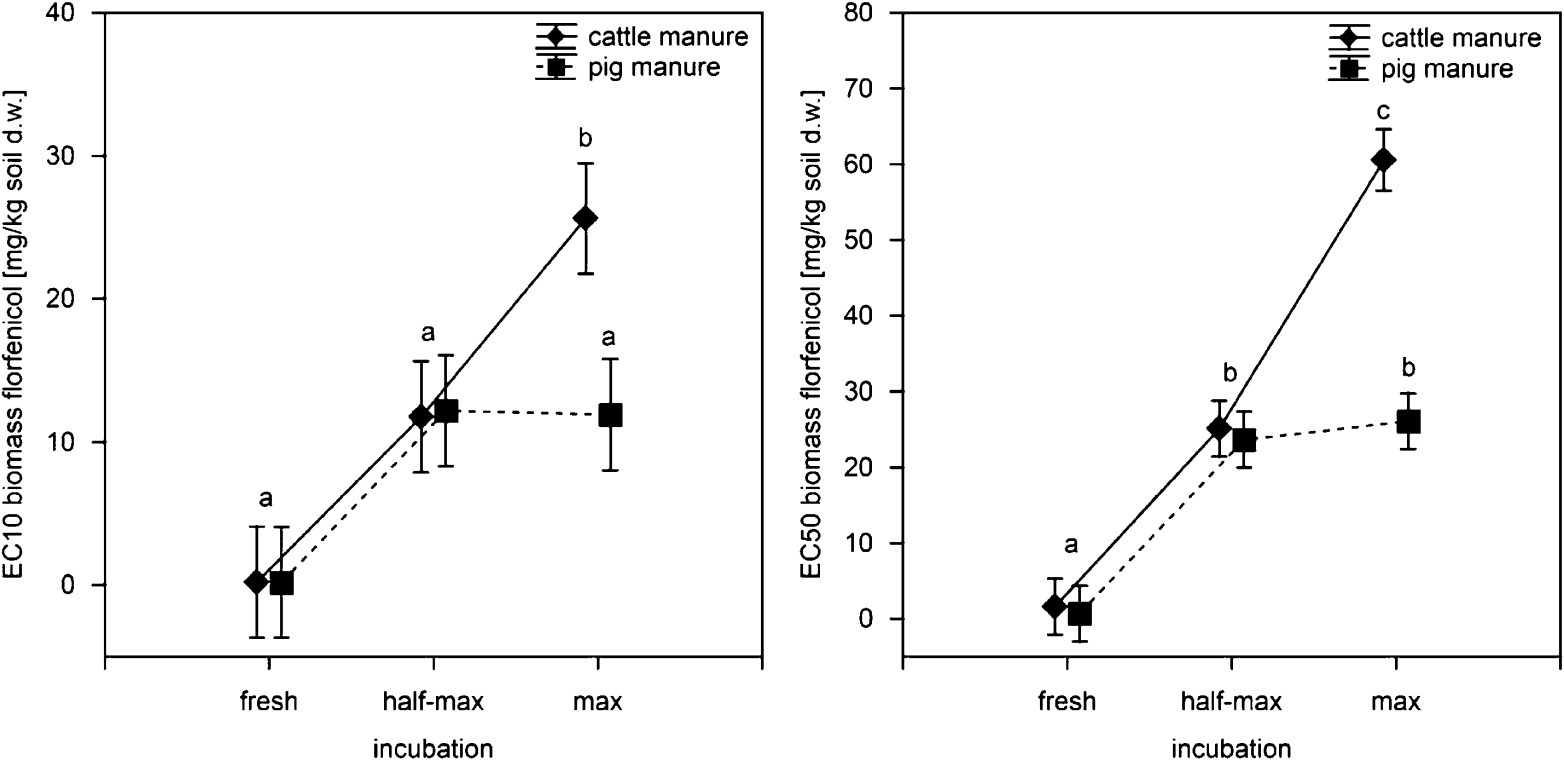
Average effect concentrations (*left* EC_10_, *right* EC_50_) for florfenicol derived from plant tests with six species in dependence on manure type and incubation time. Depicted are mean EC_*x*_ over six plant species (*A. cepa, A. sativa, B. napus, P. vulgaris, S. lycopersicum* and *S. alba*) with *bars* indicating standard error and *letters* indicating groups identified by Duncan’s test on rank order, homogenous groups, *α* = 0.05, error between MS = 90.065, df = 30)

Applying the test substance via freshly spiked manure apparently had no general influence on the effects (Figs. 2, 3), with less than factor 2 difference between retrieved EC_x_ and those from standard tests.

For florfenicol, effects in *P. vulgaris* were slightly stronger in tests with freshly spiked manure compared to standard tests, however not in any other species.

Applying florfenicol via anaerobic incubation with cattle or pig manure increased the EC_*x*_ by at least factor 10 in *B. napus* and *P. vulgaris*. The EC_10_ of florfenicol were generally more affected than the EC_50_ by the application via incubated manure. The greatest difference was observed for the EC_10_ of florfenicol in *B. napus*, which was 0.05 mg/kg soil dry mass assessed via the standard plant test and increased by factor 242 (cattle manure) and 282 (pig manure) for half-maximum and factor 540 (cattle manure) and 286 (pig manure) for maximum incubation. This trend was similar for effects of florfenicol on EC_*x*_ in *P. vulgaris* (Fig. 3).

For tylosin tartrate, the difference by manure application was generally smaller than for florfenicol, with 64 as greatest factor of difference for the EC_10_ in *P. vulgaris* from anaerobic half-maximum incubation with pig manure (Table 10). Prolonging the incubation time of tylosin tartrate in manure from half-maximum to maximum had little impact on the EC_*x*_ and seemed to follow no trend (Fig. 2). For *B. napus*, effects in tests with tylosin tartrate in incubated pig manure were too low to derive any EC_*x*_ (Table 10).

In order to examine what influence the type of manure and the incubation time had on the effect concentrations for florfenicol (not sufficient data available for tylosin tartrate), a Duncan’s test on rank order was performed on average EC_10_ and EC_50_, respectively, using the six plant species as replicates (Fig. 3). The analysis demonstrated that the type of manure (pig or cattle) was only relevant when incubated for the maximum incubation time. The period of incubation was relevant for the EC_50_ from tests with pig manure, though no influence was seen by prolonging the incubation time from half-maximum to maximum. For application via cattle manure, the EC_10_ significantly increased from fresh and half-maximum to maximum incubation time, while the EC_50_ significantly differed between all three incubation periods.

Tests on florfenicol applied via aerobic half-maximum stored manure-soil-mixture yielded substantially lower effect concentrations in *A. cepa* and *B. napus* compared to those from anaerobic incubation (see Additional file 1). The greatest difference was factor 64 for the EC_50_ (fresh mass) in *B. napus* in tests with pig manure.

In contrast, tests on tylosin tartrate applied via aerobic half-maximum stored manure-soil-mixture yielded only slightly lower effect concentrations compared to those from anaerobic incubation. The greatest difference was factor 15 for the EC_50_ (fresh mass) in *A. cepa* in cattle manure.

Thus, an aerobic storage of the spiked manure-soil-mixture did not alter the effects of the two antibiotics on shoot fresh mass when compared with the standard test design. In contrast, an anaerobic incubation of spiked pig or cattle manure resulted in significantly decreased effects of both antibiotics on shoot fresh mass.

## Discussion

### Practical aspects

In the OECD 208 test guideline [3] seedling emergence of >70 % and survival of emerged seedlings >90 % in the control is required for a test to be valid. In the presented tests, particularly the emergence rate was not always sufficient to meet this validity criterion in the species *B. napus*, *P. vulgaris, S. alba* and *S. lycopersicum*. Most likely, a too high soil moisture at test start was unfavourable for the respective plant species. However, as effect concentrations with narrow confidence intervals could be retrieved, the results are considered usable in this case.

Moreover, the elevated number of eight replicates contributed to reliable results.

The applied test item concentrations were chosen to obtain EC_50_ values. As a consequence, EC_10_ could not always be estimated satisfactorily. Effect concentrations used for a refined risk assessment in tier B, however, are usually the EC_10_. In a future test design, this aspect should be reflected by applying test item concentrations rather covering the lower effect range. This would also avoid a general handicap of test design. To achieve the nominal test concentrations in the manure/soil mixture, which are the target concentrations in the test, approx. 50-fold higher concentrations have to be applied to the manure for incubation. These high concentrations may have possible negative impact on the functioning of the manure microbial community. This could impair possible potential transformation. However, the forming of NER should be unaffected.

The applied procedure encompassing initial manure processing, storage, acclimation, and incubation of manure was shown to be suitable to maintain anaerobic conditions throughout the manure preparation phase. A manure incubation temperature of 20 °C was selected based on the temperature recommended by the guideline on determining the fate of VMPs in manure [7]. According to this guideline, degradation tests may be conducted at 20 °C as well as at 10 °C. For future tests, a temperature of 10 °C during incubation of manure should be used, which has been found to be typical for manure in a storage tank [13]. Incubation at 20 °C may cause enhanced degradation of VMPs and its TPs and lead to non-realistic residues. Overall, the presented testing approach for an extended plant test proved to be technically feasible and to result in reliable effect concentrations. The application of VMP via poultry manure was not considered in this study since requirements on aerobic conditions and dry matter content differ significantly from those for pig and cattle manure.

### Phytotoxicity of florfenicol and tylosin tartrate

Tylosin tartrate belongs to the macrolide antibiotics and acts by inhibiting bacterial protein synthesis [14, 15]. Tylosin is composed of four bioactive forms, tylosin A, B, C, and D, with tylosin A having the greatest share (80–90 %) and having the greatest activity [14, 16]. Due to poor absorption in the animal’s gut, greater than 40 % of the administered tylosin is excreted, primarily as tylosin D and tylosin A [14, 15].

Florfenicol is a fluorinated structural analog of thiamphenicol and chloramphenicol that is used as wide spectrum veterinary antibiotic and that disrupts bacterial protein synthesis [17, 18].

Little effect data from standard plant tests were available for tylosin tartrate and florfenicol at the start of the present study (ECOTOX database [19]).

In a toxicity study on tylosin tartrate according to OECD 208, the lowest EC_50_ was observed for shoot length of cucumber with 90 mg/kg soil dry weight and 35 mg/kg soil dry weight for root length [20]. This is in good agreement with the results obtained in the here presented standard tests (lowest EC_50_ for shoot length of 49.8 mg/kg soil dry mass).

For florfenicol, no effect data from standard plant tests were available in peer-reviewed journals. In the environmental assessment dossier of the product Aquaflor, however, EC_50_ values (shoot fresh mass) for florfenicol of 0.5, 1.7 and 6.7 mg/kg for cress, mustard and wheat, respectively, are reported [15]. These values are in good agreement with the ones determined in the standard tests of the present study (lowest EC_50_ for shoot fresh mass of 0.25 mg/kg soil dry mass).

### Effects of manure application

In the pre-tests, it was demonstrated that elevated rates of manure application can impair seedling emergence despite plant growth rather profiting from the nutrient supply. Cattle manure application impaired seedling emergence at application rates ≥106 kg N/ha in *P. vulgaris* while pig manure impaired seedling emergence at ≥26 kg N/ha in *T. aestivum* and at ≥106 kg N/ha in *T. pratense.*

In the extended plant tests, the validity criteria with respect to seedling emergence and seedling survival rates were not always met, mostly with pig manure application. However, this was also the case in the standard plant tests and might have been related to the test soil moisture not being ideal for all test plant species.

### Influence of anaerobic incubation conditions

Manure is usually stored in tanks were microbial processes create an anaerobic environment [13]. The presence of oxygen and the redox potential can strongly influence the degradability of chemical substances.

Anaerobic incubation of spiked manure reduced the phytotoxic effects of the two antibiotics on shoot fresh mass significantly. The difference in effect concentrations from anaerobically incubated manure and standard tests was substantially greater for florfenicol (maximum factor of 64) than for tylosin tartrate (maximum factor of 15). This indicates that the two antibiotics experienced substantial dissipation under anaerobic incubation, whereas the aerobic storage did not lead to a noticeable reduction in bioavailability or degradation of the substances. It can be concluded that anaerobic conditions promoted processes like sorption and/or degradation, chiefly in case of florfenicol. Yet, the fact that phytotoxicity still occurred demonstrates that the antibiotics (and potential transformation products) remained partially bioavailable even after an anaerobic incubation with manure for 27 and 53 days, respectively.

As demonstrated by the results, the aerobic storage of the spiked manure-soil-mixture did not yield reduced phytotoxic effects compared to the standard test design. This is surprising since laboratory studies on the fate of tylosin indicate easy degradability under aerobic conditions both in manure (half-lives of 6 and 8 days for cattle and pig excreta, respectively) [14] and in soil (half-live of 7–8 days) [20].

Besides degradation, disappearance of tylosin in manure is attributed to irreversible sorption, i.e. the formation of non-extractable bound residues [21]. In contrast to our results, faster disappearance rates were noticed in aerated than in non-aerated samples [21].

Given a (calculated) log Kow of 2.5, the unspecific binding of the uncharged tylosin molecule to organic carbon should be moderate [22]. The pKa of tylosin is around 7.7 so that in manure with a basic pH the substance is present both as the positively charged cation and the uncharged molecule [23]. With decreasing pH, the degree of protonation and the binding to negatively charged matrices such as manure and soil increases [23, 24]. Yet, Loke et al. [23] found that instead of ionic binding sorption of tylosin (precisely tylosin A) in manure is mainly caused by hydrophobic interactions with organic matter. Apparently, sorption can substantially delay degradation of tylosin. In spiked manure, residual tylosin was detected after 8 months of incubation [21]. Similarly, a prolonged half-life for tylosin A of 49 days was determined in a sandy field soil [22] and of 95 and 97 days in a sandy loam and a clay loam, respectively [25]. Hence, despite its biodegradability, tylosin can be persistent in field soils where dissipation may be much slower than under laboratory conditions.

Florfenicol is readily water soluble and considered to be a weakly hydrophobic substance due to its low log Kow (−0.04) and Koc (24–52 L/kg) values [26, 27]. Thus, its binding potential to organic matter can be considered low. Amending soil with manure can further decrease florfenicol sorption to soil by competition of florfenicol with dissolved organic matter at the available soil surface [26]. Degradability of florfenicol was demonstrated in aerobic cattle manure as well as in anaerobic pig manure [15]. Against this background it is surprising that phytotoxicity was not reduced after the aerobic incubation in the present study.

Florfenicol is biodegradable in soil with reported half-lives of 8–73 days under laboratory conditions [15]. However, degradation time increases with increasing initial florfenicol concentration, probably due to inhibition of microbial activity [15]. In field soil, however, half-lives for florfenicol of greater than 103 days were determined [26].

To summarise, both antibiotics are biodegradable under laboratory conditions with delayed degradation in field soils. Whereas tylosin tartrate has a strong sorption potential, florfenicol should be rather present in the soil solution. This may be the cause why the observed impact of anaerobic incubation on phytotoxicity was greater for florfenicol.

### Influence of incubation period

Manure is usually stored between 1 and 4 months before it is distributed on land [28]. To quantify the influence of the storage duration, the test substance spiked manure was applied to soil either fresh, after a half-maximum or after a maximum incubation period under anaerobic conditions.

The application of the two antibiotics via freshly spiked manure did not alter their phytotoxic effects substantially compared to the standard test. For tylosin, this may be attributed to only slightly higher distribution coefficient (Kd) values in manure (36–295 L/kg [23]) compared to soils (8–128 L/kg [29]).

Concerning tylosin tartrate, effect concentrations from half-maximum incubated pig manure exceeded those from standard tests by up to a factor of 64. Prolonging the incubation period did not lead to a further increase of the effect concentrations. The effect of incubation was generally greater for florfenicol than for tylosin tartrate with effect concentrations from maximum incubated cattle manure exceeding those from standard tests by a factor of 540. While for pig manure, no influence of a prolonged incubation was discernible, for florfenicol spiked cattle manure phytotoxicity significantly decreased between the half-maximum and the maximum incubation period.

One reason for the different impact of cattle and pig manure could be the higher organic matter content in cattle manure (approximately 10 % compared to 5 % in pig manure) as it represents the surface for non-specific sorption. Another reason could be the incubation period which was not identical for the two manure types: maximum and half-maximum incubation time for pig manure was 27 and 53 days, respectively, while for cattle manure, it was 45 and 90 days, respectively. Thus, the greater impact of applying the test substance via cattle than via pig manure is probably due to both the elevated organic matter content of and the longer incubation time in cattle manure.

### Implication of findings and realism of test design

For a plant risk assessment, the predicted environmental concentrations (PEC) in soil calculated according to the CVMP guideline [2] is compared to the predicted no effect concentration (PNEC) obtained from the seedling emergence and seedling growth test according to OECD 208 after applying an assessment factor (AF) of 100. In tier B, the NOEC or EC_10_ is multiplied with an AF of 10. If a risk for plants is identified, the EMA reflection paper [4] recommends conducting a higher tier assessment by applying a statistical extrapolation technique, the so-called species sensitivity distribution (SSD). The extended plant test would add the option to conduct a modified test in case a risk for plants has been identified and the substance has been shown to form non-extractable residues in manure or transformation products ≥10 % of the applied amount.

In the presented extended plant tests on tylosin tartrate and florfenicol, phytotoxicity was significantly reduced compared to the standard plant test procedure when applying spiked manure that had been incubated at 20 °C. Tylosin tartrate and florfenicol were selected because they are veterinary antibiotics, marketing authorisation applications have been submitted in recent years and they are known to form transformation products including high amounts of non-extractable residues.

Effect concentrations (EC_10_) were up to three orders of magnitude higher by (up to factor 64 for tylosin tartrate and 540 for florfenicol) when applying via manure compared to EC_10_ derived in standard tests.

Spiking manure with the VMPs is a more realistic exposure way than applying it directly to soil; however, the potential formation of metabolites in the treated animal is not accounted for by this approach. Degradation of tylosin tartrate has been studied using manure from animals administered the particular antibiotic [30]; however, plant tests are not reported so far.

Although the applied extended testing approach represents a more realistic exposure scenario, it does not cover all potential risks from VMPs toward plants. For example, leaching may be stronger in the field where rain can wash soluble and manure particle bound substances in deeper soil horizons. Higher plant density may promote microbial degradation in the rhizosphere. Antibiotics can be taken up into plants with reduced soil concentrations as consequence. Whereas no uptake of tylosin tartrate into plants grown in manure-added soil was found [31], florfenicol was detected in plants at concentrations reaching 38 µg/kg in carrot peel (grown in 1 mg/kg soil dry weight spiked soil for 152 days [26]).

Yet, microbial degradation during incubation of manure may be much lower in the winter and spring season than in summer. Spreading manure on plant tissue may cause higher damage than mixing manure into soil and adding plant seeds afterwards. Non-crop species may be more sensitive to VMPs than the tested crop species. Anaerobic conditions may inhibit rather than promote the degradation of some VMPs. The occurrence of not one but various VMPs may lead to combined effects. In these cases, the obtained results would overestimate the mitigating effect of incubated manure as exposure matrix.

At present, there appears to be no similar studies on developing an extended, more realistic plant test approach for assessing the phytotoxicity of VMPs.

## Conclusions

This study was conducted to develop a testing strategy for plants in the tier B assessment. The verification of the extended plant test showed that seedling emergence and growth are comparable to a standard OECD test and reliable effect concentrations could be established. Appropriate growth conditions were verified for pig and cattle manure up to a maximum manure concentration representing 85 kg N/ha (half-maximum amount allowed per year in Europe), at least for the seven plant species *A. cepa*, *A. sativa*, *S. lycopersicum*, *B. napus*, *P. vulgaris*, *S. alba*, and *T. pratense*, representing monocots, dicots, and legumes. As demonstrated in the present study, phytotoxicity of veterinary antibiotics can be significantly reduced by application via incubated manure compared to the standard plant tests.

On the basis of the presented study, an operation manual for a standardised extended plant test is under preparation. Further, the presented approach will be included in an updated EMA guideline on the plant testing strategy for VMPs which is currently under preparation.

## Additional file

10.1186/s12302-016-0089-2 Additional tables (details on plant species, test conditions and EC_x_ values of the different plant tests with manure).

## Abbreviations

API: active pharmaceutical ingredient
ECx: effect concentration causing x % inhibition compared to control
EMA: European Medicines Agency
ERA: environmental risk assessment
ME: metabolite
NER: non-extractable residues
NOEC: no observed effect concentration
OECD: Organisation for Economic Co-operation and Development
TP: transformation product
VMP: veterinary pharmaceutic product
