# Supplementary material for: Results of extended plant tests using more realistic exposure scenarios for improving environmental risk assessment of veterinary pharmaceuticals
Source: Environ Sci Eur. 2016 Aug 9;28(1):22. doi: 10.1186/s12302-016-0089-2 (PMC5044973; doi:10.1186/s12302-016-0089-2)
Supplement: Supplementary file 1 — 10.1186/s12302-016-0089-2 Additional tables (details on plant species, test conditions and ECx values of the different plant tests with manure). [file 12302_2016_89_MOESM1_ESM.pdf]

Table S1: Information on cultivar and supplier of plant seeds used in the tests:

Species	Cultivar	Supplier
Allium cepa	Stuttgarter Riesen	Enza Zaden Deutschland GmbH & Co. KG, Germany
Triticum aestivum	Kredo	Nordsaat, Saatzuchtgesellschaft mbH, Granskevitz, Germany
Avena sativa	Champion	Nordsaat, Saatzuchtgesellschaft mbH, Granskevitz, Germany
Solanum lycopersicum	Moneyberg	Enza Zaden Deutschland GmbH & Co. KG, Germany
Brassica napus	Liforum	Lufa Speyer, Speyer, Deutschland.
Sinapis alba	Litember	Bruno Nebelung GmbH, Everswinkel, Deutschland
Cucumis sativus	Delikateß	Enza Zaden Deutschland GmbH & Co. KG, Germany
Phaseolus vulgaris	Primel	Enza Zaden Deutschland GmbH & Co. KG, Germany
Trifolium pratense	Nemaro	Bruno Nebelung GmbH, Everswinkel, Germany

Table S2: Test conditions for plant tests with different manure concentrations.

	A. cepa	***B. napus***	***C. sativus***	***P. vulgaris***	***S. alba***	***S. lycopersicum***	***T. aestivum***	***T. pratense***
seeds per pot	6	5	3	5	5	5	6	6
replicates (pots) per treatment	4	4 (5 in C)	6	4	4 (5 in C)	4 (5 in C)	4	4
total number of seeds	24	20 (25 in C)	18	20	20 (25 in C)	20 (25 in C)	24	24
application range	7, 11, 18, 29 and 46 g manure fresh mass per kg soil dry mass (approximately 26, 42, 66, 106 and 170 kg N/ha)							
control(s)	standard control							
climatic conditions (target values)								
temperature range [°C]	22 ± 10							
air humidity [%]	70 % ± 25 %							
light intensity [µE/m2*s]	>200							

Table S3: Test conditions for plant tests with florfenicol.

**Florfenicol**						
	A. cepa	***A. sativa***	***B. napus***	***S. alba***	***S. lycopersicum***	***P. vulgaris***
**standard test**						
seeds per pot	5	5	5	5	5	4
replicates (pots) per treatment	4	4	4	4	4	5
total number of seeds	20	20	20	20	20	20
application range [mg/kg soil dry mass]	0.06 - 5.0 (all species)					
control(s)	standard control (4 replicates), solvent control (4 replicates)					
temperature range [°C]	16 - 32					
air humidity [%]	25 - 88					
light intensity [µE/m2*s]	280 - 400					
chemical analysis	85% recovery in spiked soil at representative soil concentrations					
**fresh spiked pig manure**	***A. cepa***	***A. sativa***	***B. napus***	***S. alba***	***S. lycopersicum***	***P. vulgaris***
seeds per pot	5	5	5	5	5	4
replicates (pots) per treatment	8	8	8	8	8	8
total number of seeds	40	40	40	40	40	32
application range [mg/kg soil dry mass]	0.2 - 16.7					
control(s)	standard control (4 - 5 replicates with each 20 seeds), manure solvent control (8 replicates)					
temperature range [°C]	19 - 28					
air humidity [%]	35 -70					
light intensity [µE/m2*s]	135 - 242					
chemical analysis	85% - 95% recovery in highest application solution and 93% in spiked manure					
**fresh spiked cattle manure**	***A. cepa***	***A. sativa***	***B. napus***	***S. alba***	***S. lycopersicum***	***P. vulgaris***
seeds per pot	5	5	5	5	5	4
replicates (pots) per treatment	8	8	8	8	8	8
total number of seeds	40	40	40	40	40	32
application range [mg/kg soil dry mass]	0.2 - 16.7					
control(s)	standard control (4 - 5 replicates with each 20 seeds), manure solvent control (8 replicates)					
temperature range [°C]	19 - 28					
air humidity [%]	35 -70					
light intensity [µE/m2*s]	135 - 242					
chemical analysis	94% - 110% recovery in highest application solution and 98% in spiked manure					

Table S3 (continued):

**half-max incubated pig manure**	***A. cepa***	***A. sativa***	***B. napus***	***S. alba***	***S. lycopersicum***	***P. vulgaris***
seeds per pot	5	5	5	5	5	4
replicates (pots) per treatment	8	8	8	8	8	8
total number of seeds	40	40	40	40	40	32
application range [mg/kg soil dry mass]	0.62 - 50					
control(s)	standard control (4 - 5 replicates with each 20 seeds), manure solvent control (8 replicates)					
temperature range [°C]	16 - 28					
air humidity [%]	35 - 65					
light intensity [µE/m2*s]	212 - 445					
chemical analysis	94% - 100% recovery in highest application solution and 4% in spiked manure					
**half-max incubated cattle manure**	***A. cepa***	***A. sativa***	***B. napus***	***S. alba***	***S. lycopersicum***	***P. vulgaris***
seeds per pot	5	5	5	5	5	4
replicates (pots) per treatment	8	8	8	8	8	8
total number of seeds	40	40	40	40	40	32
control(s)	standard control (4 - 5 replicates with each 20 seeds), manure solvent control (8 replicates)					
application range [mg/kg soil dry mass]	0.62 - 50					
temperature range [°C]	18 - 24					
air humidity [%]	28 - 55					
light intensity [µE/m2*s]	110 - 293					
chemical analysis	103% - 104% in highest application solution and 3% in spiked manure					
**max incubated pig manure**	***A. cepa***	***A. sativa***	***B. napus***	***S. alba***	***S. lycopersicum***	***P. vulgaris***
seeds per pot	5	5	5	5	5	4
replicates (pots) per treatment	8	8	8	8	8	8
total number of seeds	40	40	40	40	40	32
application range [mg/kg soil dry mass]	0.62 - 50					
control(s)	standard control (4 - 5 replicates with each 20 seeds), manure solvent control (8 replicates)					
temperature range [°C]	16 - 28					
air humidity [%]	35 - 65					
light intensity [µE/m2*s]	212 - 445					
chemical analysis	94% - 100% recovery in highest application solution and 2% in spiked manure					
**max incubated cattle manure**	***A. cepa***	***A. sativa***	***B. napus***	***S. alba***	***S. lycopersicum***	***P. vulgaris***
seeds per pot	5	5	5	5	5	4
replicates (pots) per treatment	8	8	8	8	8	8
total number of seeds	40	40	40	40	40	32
application range [mg/kg soil dry mass]	0.62 - 50					
control(s)	standard control (4 - 5 replicates with each 20 seeds), manure solvent control (8 replicates)					
temperature range [°C]	18 - 27					
air humidity [%]	25 - 50					
light intensity [µE/m2*s]	183 - 319					
chemical analysis	103% - 104% recovery in highest application solution and 3% in spiked manure					

Table S4: Test conditions for plant tests with tylosin tartrate.

**Tylosin tartrate**						
	A. cepa	***A. sativa***	***B. napus***	***S. lycopersicum***	***P. vulgaris***	***T. pratense***
**standard test**						
seeds per pot	*5*	*5*	*5*	*5*	*3*	*5*
replicates (pots) per treatment	4	4	4	4	4	4
total number of seeds	20	20	20	20	12	20
application range [mg/kg soil dry mass]	27.8 – 250	50.0 – 800	16.0 – 144	36.1 – 144	50.0 – 800	9.3 – 83.3
control(s)	standard control (4 replicates)					
temperature range [°C]	22 - 24					
air humidity [%]	38 - 50					
light intensity [µE/m2*s]	>200					
chemical analysis	no analysis					
**fresh spiked pig manure**	***A. cepa***	***A. sativa***	***B. napus***	***S. lycopersicum***	***P. vulgaris***	***T. pratense***
seeds per pot	*5*	*5*	*5*	*5*	*3*	*5*
replicates (pots) per treatment	8	8	8	8	8	8
total number of seeds	40	40	40	40	24	40
application range [mg/kg soil dry mass]	15 - 300	100 – 1000	5 - 391	5 - 391	40 – 937	5 - 125
control(s)						
temperature range [°C]	21 - 24					
air humidity [%]	39 - 69					
light intensity [µE/m2*s]	>200					
chemical analysis	6.0% recovery in spiked manure					
**fresh spiked cattle manure**	***A. cepa***	***A. sativa***	***B. napus***	***S. lycopersicum***	***P. vulgaris***	***T. pratense***
seeds per pot	*5*	*5*	*5*	*5*	*3*	*5*
replicates (pots) per treatment	8	8	8	8	8	8
total number of seeds	40	40	40	40	24	40
application range [mg/kg soil dry mass]	15 - 300	100 – 1000	5 - 391	5 - 391	40 – 937	5 - 125
control(s)	standard control (4 replicates), manure control (8 replicates)					
temperature range [°C]	17 - 28					
air humidity [%]	23 - 62					
light intensity [µE/m2*s]	>200					
chemical analysis	6.5% recovery in spiked manure					

Table S4 (continued):

**half-max incubated pig manure**	***A. cepa***	***A. sativa***	***B. napus***	***S. lycopersicum***	***P. vulgaris***	***T. pratense***
seeds per pot	*5*	*5*	*5*	*5*	*3*	*5*
replicates (pots) per treatment	8	8	8	8	8	8
total number of seeds	40	40	40	40	24	40
application range [mg/kg soil dry mass]	15 - 300	100 - 1000	15 - 300	15 - 300	40 - 937	5 - 391
control(s)	standard control (4 replicates), manure control (8 replicates)					
temperature range [°C]	21 - 26					
air humidity [%]	35 - 70					
light intensity [µE/m2*s]	>200					
chemical analysis	8.1% recovery in spiked manure					
**half-max incubated cattle manure**	***A. cepa***	***A. sativa***	***B. napus***	***S. lycopersicum***	***P. vulgaris***	***T. pratense***
seeds per pot	*5*	*5*	*5*	*5*	*3*	*5*
replicates (pots) per treatment	8	8	8	8	8	8
total number of seeds	40	40	40	40	24	40
control(s)	standard control (4 replicates), manure control (8 replicates)					
application range [mg/kg soil dry mass]	15 - 300	100 – 1000	5 - 391	5 - 391	40 – 937	5 - 125
temperature range [°C]	16 - 26					
air humidity [%]	27 - 52					
light intensity [µE/m2*s]	>200					
chemical analysis	17% recovery in spiked manure					
**max incubated pig manure**	***A. cepa***	***A. sativa***	***B. napus***	***S. lycopersicum***	***P. vulgaris***	***T. pratense***
seeds per pot	*5*	*5*	*5*	*5*	*3*	*5*
replicates (pots) per treatment	8	8	8	8	8	8
total number of seeds	40	40	40	40	24	40
application range [mg/kg soil dry mass]	15 - 300	100 – 1000	5 - 391	5 - 391	40 – 937	5 - 125
control(s)	standard control (4 replicates), manure control (8 replicates)					
temperature range [°C]	17 - 25					
air humidity [%]	25 - 59					
light intensity [µE/m2*s]	>200					
chemical analysis	11.4% recovery in spiked manure					

Table S4 (continued):

**max incubated cattle manure**	***A. cepa***	***A. sativa***	***B. napus***	***S. lycopersicum***	***P. vulgaris***	***T. pratense***
seeds per pot	*5*	*5*	*5*	*5*	*3*	*5*
replicates (pots) per treatment	8	8	8	8	8	8
total number of seeds	40	40	40	40	24	40
application range [mg/kg soil dry mass]	15 - 300	100 – 1000	5 - 391	5 - 391	40 – 937	5 - 125
control(s)	standard control (4 replicates), manure control (8 replicates)					
temperature range [°C]	21 - 25					
air humidity [%]	18 - 44					
light intensity [µE/m2*s]	>200					
chemical analysis	14.9% recovery in spiked manure					

Table S5: Effect concentrations (EC_{10}, EC_{50} with 95% confidence interval) and NOEC, in kg N/ha, for pre-tests with cattle manure.

**Species**	***A. cepa***	***T. aestivum***	***B. napus***	***S. lycopersicum***	***P. vulgaris***	***T. pratense***
Emergence						
NOEC	≥ 170	≥ 170	≥ 170	≥ 170	≥ 170	≥ 170
EC_{10}	28.1 (n.d.)	n.d.	477 (n.d.)	n.d.	n.d.	53.1
EC_{50}	n.d. ≥ 170	n.d. ≥ 170	n.d. ≥ 170	n.d. ≥ 170	n.d. ≥ 170	n.d. ≥ 170
Post-emergence survival						
NOEC	≥ 170	≥ 170	≥ 170	≥ 170	≥ 170	≥ 170
EC_{10}	n.d.	n.d.	n.d.	n.d.	n.d.	115
EC_{50}	n.d.	n.d.	n.d.	n.d.	n.d.	n.d.
Shoot length						
NOEC	≥ 170	106	≥ 170	≥ 170	-	≥ 170
EC_{10}	n.d.	n.d.	n.d.	n.d.	-	n.d.
EC_{50}	n.d.	n.d.	n.d.	n.d.	-	n.d.
Fresh mass						
NOEC	≥ 170	≥ 170	≥ 170	≥ 170	≥ 170	≥ 170
EC_{10}	n.d.	n.d.	n.d.	n.d.	n.d.	n.d.
EC_{50}	n.d.	n.d.	n.d.	n.d.	n.d.	n.d.
valid:	yes	yes	yes	yes	yes	yes

Table S6: Effect concentrations (EC_{10}, EC_{50} with 95% confidence interval) and NOEC, in kg N/ha, for pre-tests with pig manure.

**Species**	***A. cepa***	***T. aestivum***	***B. napus***	***S. lycopersicum***	***P. vulgaris***	***C. sativus***	***S. alba***	***T. pratense***
Emergence								
NOEC	≥ 170	≥ 170	≥ 170	102	≥ 170	102	≥ 170	≥ 170
EC_{10}	n.d.	n.d.	4.40	82.5	44.9	42.18	29.2	n.d.
					(1.8 - 76.4	(16.2 - 61.1)	n.d.	
EC_{50}	n.d.	n.d.	n.d.	200	n.d.	166	327	n.d.
						(114 - 451)	n.d.	
Post-emergence survival								
NOEC	≥ 170	≥ 170	≥ 170	≥ 170	≥ 170	≥ 170	≥ 170	≥ 170
EC_{10}	n.d.	n.d.	114	n.d.	n.d.	n.d.	n.d.	n.d.
			(n.d.)					
EC_{50}	n.d.	n.d.	n.d.	n.d.	n.d.	n.d.	n.d.	n.d.
Shoot length								
NOEC	≥ 170	≥ 170	≥ 170	≥ 170	≥ 170	≥ 170	102	≥ 170
EC_{10}	n.d.	n.d.	n.d.	n.d.	n.d.	n.d.	79.9	n.d.
EC_{50}	n.d.	n.d.	n.d.	n.d.	n.d.	n.d.	360	n.d.
Fresh mass								
NOEC	≥ 170	≥ 170	≥ 170	≥ 170	≥ 170	≥ 170	≥ 170	≥ 170
EC_{10}	n.d.	n.d.	n.d.	n.d.	n.d.	n.d.	n.d.	n.d.
EC_{50}	n.d.	n.d.	n.d.	n.d.	n.d.	n.d.	n.d.	n.d.
	yes	yes	yes	yes	yes	no (83% emerged, 80% survived)	no (76% emerged, 79 survived)	yes

Table S7: Effect concentrations (EC_{10}, EC_{50} with 95% confidence interval and NOEC, in mg/kg soil dry mass) for **florfenicol applied via freshly spiked pig manure**.

**Species**	***A. cepa***	***A. sativa***	***B. napus***	***S. alba***	***S. lycopersicum***	***P. vulgaris***
Emergence						
NOEC	≥ 16.7	≥ 16.7	≥ 16.7	≥ 16.7	5.6	0.6
EC_{10}	4.77	n.d.	0.69	3.53	3.54	0.18
	(2.11 - 9.04)	(-)	(0.06 - 1.75)	(1.41 - 6.92)	(n.d.)	(n.d.)
EC_{50}	n.d.	n.d.	n.d.	n.d.	10.62	15.18
	(-)	(-)	(-)	(-)	(n.d.)	(n.d.)
Post-emergence survival						
NOEC	0.6	5.6	0.2	0.6	0.2	1.9
EC_{10}	0.13*	7.94	0.21	0.61	0.12*	2.35
	(0.05 - 0.21)	(2.54 - 202)	(n.d.)	(0.39 - 0.80)	(0.03 - 0.23)	(n.d.)
EC_{50}	0.44	n.d.	0.48	1.35	0.8	5.52
	(0.30 - 0.63)	(-)	(n.d.)	(1.07 - 1.70)	(0.49 - 1.26)	(n.d.)
Shoot length						
NOEC	0.6	0.2	< 0.2	< 0.2	0.2	0.2
EC_{10}	0.48	0.6	n.d.	n.d.	0.17*	0.38
	(n.d.)	(0.05 - 1.45)	(-)	(-)	(n.d.)	(0.02 - 0.93)
EC_{50}	0.79	8.9	0.48	0.43	0.92	2.86
		(5.04 - 23.7)	(n.d.)	(n.d.)	(n.d.)	(1.34 - 6.26)
Fresh mass						
NOEC	≥ 1.9	0.2	< 0.2	< 0.2	0.2	< 0.2
EC_{10}	0.43	0.15	0.08*	0.08*	0.33	0.07*
	(0.42 - 0.44)	(0.02 - 0.35)	(0.07 - 0.09)	(n.d.)	(n.d.)	(0.00 - 0.27)
EC_{50}	0.73	1.37	0.21	0.22	0.47	1.0
	(0.72 - 0.74)	(0.73 - 2.49)	(0.21 - 0.22)	(n.d.)	(n.d.)	(0.26 - 2.77)
valid:	no (65% emerged, 62% survived)	yes	no (67% emerged, 60% survived)	valid	valid	no (80% emerged, 88% survived)
application range: 0.2 - 16.7 mg test item/kg dry mass soil						

* indicated value was extrapolated beyond test concentrations.

Table S8: Effect concentrations (EC_{10}, EC_{50} with 95% confidence interval and NOEC, in mg/kg soil dry mass) for **florfenicol applied via freshly spiked cattle manure**.

**Species**	***A. cepa***	***A. sativa***	***B. napus***	***S. alba***	***S. lycopersicum***	***P. vulgaris***
Emergence						
NOEC	≥ 16.7	≥ 16.7	≥ 16.7	≥ 16.7	≥ 16.7	≥ 16.7
EC_{10}	12.2	n.d.	3.6	n.d.	8.04	1.2
	(4.58 - 421)	(-)	(n.d.)	(-)	(n.d.)	(0.43 - 2.15)
EC_{50}	n.d.	n.d.	n.d.	n.d.	26.6	17.83
	(-)	(-)	(-)	(-)	(n.d.)	(9.57 - 57.7)
Post-emergence survival						
NOEC	0.60	≥ 16.7	0.20	0.20	0.20	1.90
EC_{10}	0.41	16.25	0.35	0.30	0.22	1.24
	(0.22 -0.63)	(n.d.)	(0.21 - 0.49)	(0.17 - 0.44)	(0.04 - 0.48)	(0.26 - 2.71)
EC_{50}	1.75	n.d.	1.04	1.13	4.66	21.33
	(1.27 - 2.43)	(-)	(0.80 - 1.36)	(0.83 - 1.53)	(2.64 - 11.3)	(8.26 - 284)
Shoot length						
NOEC	0.20	0.20	< 0.2	< 0.2	0.20	0.60
EC_{10}	0.46	1.52	0.08*	0.09*	0.43	0.60
	(n.d.)	(0.15 - 3.16)	(n.d.)	(n.d.)	(0.18 - 0.72)	(0.04 - 1.32)
EC_{50}	1.31	30.3	0.61	0.16*	2.79	3.31
	(0.26 - 10.5)	(15.6 - 217)	(n.d.)	(n.d.)	(2.04 - 3.84)	(1.65 - 3.33)
Fresh mass						
NOEC	0.20	0.20	< 0.2	< 0.2	0.20	< 0.2
EC_{10}	0.30	0.56	0.07*	0.06*	0.24	0.08*
	(0.16 - 0.41)	(0.18 - 1.03)	(n.d.)	(0.04 - 0.08)	(0.22 - 0.26)	(0.03 - 0.16)
EC_{50}	0.84	6.34	0.2	0.22	0.96	1.18
	(0.67 - 1.06)	(4.46 - 9.70)	(n.d.)	(0.20 - 0.25)	(0.92 - 1.00)	(0.81 - 1.67)
valid:	yes	yes	yes	yes	yes	yes
application range: 0.2 - 16.7 mg test item/kg dry mass soil						

* indicated value was extrapolated beyond test concentrations.

Table S9: Effect concentrations (EC_{10}, EC_{50} with 95% confidence interval and NOEC, in mg/kg soil dry mass) for **florfenicol applied via half-maximum incubation with pig manure**.

Species	*A. cepa*	*A. sativa*	*B. napus*	*S. alba*	*S. lycopersicum*	*P. vulgaris*
Emergence						
NOEC	≥ 50	≥ 50	≥ 50	≥ 50	16.7	≥ 50
EC_{10}	6.34	n.d.	85.6	13.2	5.50	22.5
	(1.30 - 16.4)	(-)	(n.d.)	(n.d.)	(n.d.)	(15.2 - 29.6)
EC_{50}	n.d.	n.d.	n.d.	n.d.	29.6	62.2
	(-)	(-)	(-)	(-)	(n.d.)	(45.2 - 112)
Post-emergence survival						
NOEC	16.7	16.7	16.7	16.7	16.7	16.7
EC_{10}	7.93	31.9	3.33	11.9	16.9	8.62
	(n.d.)	(n.d.)		(n.d.)	(n.d.)	(2.24 - 20.1)
EC_{50}	59.9	n.d.	30.2	32.3	42.0	109
	(n.d.)	(n.d.)	(n.d.)	(n.d.)	(n.d.)	(n.d.)
Shoot length						
NOEC	5.60	16.7	5.60	5.60	16.7	5.60
EC_{10}	11.9	7.18	15.1	14.8	20.1	15.2
	(3.83 - 17.8)	(n.d.)	(10.7 - 18.7)	(10.7 - 18.2)	(12.6 - 25.5)	(13.2 - 17.0)
EC_{50}	29.9*	40.4	29.2	27.5	31.4	33.0
	(21.1 - 42.4)	(n.d.)	(24.5 - 34.6)	(23.1 - 33.3)	(24.5 - 36.9)	(30.9 - 35.1)
Fresh mass						
NOEC	5.6	16.7	16.7	1.9	16.7	16.7
EC_{10}	5.43	18.9	14.1	10.2	12.7	11.8
	(0.17 - 10.1)	(18.7 - 19.0)	(14.0 - 14.2)	(n.d.)	(0.0 - 22.9)	(n.d.)
EC_{50}	17.5*	29.7	22.5	20.0	25.3	27.0
	(8.83 - 35.6)	(29.5 - 29.8)	(22.3 - 22.7)	(n.d.)	(n.d.)	(n.d.)
valid:	no (70% emerged, 86% survived)	yes	yes	yes	yes	yes
application range: 0.62 - 50.0 mg test item/kg dry mass soil						

* indicated value was extrapolated beyond test concentrations.

Table S10: Effect concentrations (EC_{10}, EC_{50} with 95% confidence interval and NOEC, in mg/kg soil dry mass) for **florfenicol applied via half-maximum incubation with cattle manure**.

**Species**	***A. cepa***	***A. sativa***	***B. napus***	***S. alba***	***S. lycopersicum***	***P. vulgaris***
Emergence						
NOEC	≥ 50	≥ 50	≥ 50	≥ 50	16.7	≥ 50
EC_{10}	n.d.	n.d.	n.d.	52.4	11.5	33.8
	(-)	(-)	(-)	(n.d.)	(n.d.)	(n.d.)
EC_{50}	n.d.	n.d.	n.d.	n.d.	26.6	116
	(-)	(-)	(-)	(-)	(n.d.)	(n.d.)
Post-emergence survival						
NOEC	16.7	≥ 50	16.7	16.7	16.7	≥ 50
EC_{10}	12.3	n.d.	5.67	22.2	20.6	39.6
	(9.36 - 15.2)	(-)	(n.d.)	(n.d.)	(n.d.)	(n.d.)
EC_{50}	25.4	n.d.	23.5	32.6	51.5*	85.3*
	(20.9 - 31.8)	(-)	(n.d.)	(n.d.)	(n.d.)	(n.d.)
Shoot length						
NOEC	≥ 16.7	16.7	5.60	1.90	5.60	16.7
EC_{10}	10.7	23.1	12.5	15.6	15.6	17.6
	(10.6 - 10.7)	(n.d.)	(10.9 - 13.4)	(4.91 - 22.5)	(13.8 - 17.2)	(17.4 -17.7)
EC_{50}	37.8	42.4	33.9	27.6	26.7	34.8
	(37.3 - 38.4)	(n.d.)	(28.4 - 47.3)	(18.1 - 49.1)	(24.5 - 29.4)	(34.6 - 34.9)
Fresh mass						
NOEC	5.60	16.7	5.60	1.90	16.7	5.60
EC_{10}	7.71	14.2	12.1	8.10	16.7	11.8
	(7.35 - 8.04)	(0.10 - 25.1)	(n.d.)	(n.d.)	(16.6 - 16.8)	(9.14 - 14.2)
EC_{50}	26.5	29.4	20.8	21.1	24.2	28.7
	(25.5 - 27.5)	(12.2 - 162)	(n.d.)	(n.d.)	(23.8 - 24.6)	(25.6 - 32.3)
valid:	yes	yes	yes	no (55% emerged, 100% survived)	no (85% emerged, 82% survived)	yes
application range: 0.62 - 50.0 mg test item/kg dry mass soil						

* indicated value was extrapolated beyond test concentrations.

Table S11: Effect concentrations (EC_{10}, EC_{50} with 95% confidence interval and NOEC, in mg/kg soil dry mass) for **florfenicol applied via maximum incubation with pig manure**.

**Species**	***A. cepa***	***A. sativa***	***B. napus***	***S. alba***	***S. lycopersicum***	***P. vulgaris***
Emergence						
NOEC	≥ 50	≥ 50	≥ 50	≥ 50	16.7	16.7
EC_{10}	n.d.	n.d.	n.d.	n.d.	18.3	1.33
	(-)	(-)	(-)	(-)	(n.d.)	(0.01 - 4.29)
EC_{50}	n.d.	n.d.	n.d.	n.d.	46.1	501
	(-)	(-)	(-)	(-)	(n.d.)	(n.d.)
Post-emergence survival						
NOEC	16.7	≥ 50	16.7	16.7	1.9	16.7
EC_{10}	2.08	n.d.	5.77	6.87	18.4	17.0
	(0.46 - 4.19)	(-)	(n.d.)	(2.72 - 11.9)	(n.d.)	(5.82 - 27.8)
EC_{50}	18.47	n.d.	22.32	95*	37.7	73.3
	(9.8 - 56.4)	(-)	(n.d.)	(45.8 - 492)	(n.d.)	(41.4 - 508)
Shoot length						
NOEC	16.7	16.7	≥ 16.7	5.6	1.9	16.7
EC_{10}	8.71	14.06	17.44	8.34	16.0	29.3
	(n.d.)	(n.d.)	(n.d.)	(6.24 - 10.1)	(4.64 - 23.5)	(25.2 - 32.3)
EC_{50}	33.74	n.d.	n.d.	20.9	30.5	43.9
	(n.d.)	(-)	(-)	(18.7 - 23.5)	(19.2 - 44.4)	(42.3 - 44.9)
Fresh mass						
NOEC	16.7	16.7	≥ 16.7	5.6	1.9	16.7
EC_{10}	0.4*	16.8	14.3	9.44	2.35	28.2
	(n.d.)	(16.6 -17.0)	(n.d.)	(5.88 - 11.3)	(n.d.)	(24.2 - 32.2)
EC_{50}	19.8	37.2	27.8	15.7	14.8	41.2
	(n.d.)	(37.0 - 37.4)	(n.d.)	(14.8 - 16.1)	(n.d.)	(39.1 - 42.6)
valid:	no (80% emerged, 81% survived)	valid	no (80% emerged, 81% survived)	valid	valid	valid
application range: 0.62 - 50.0 mg test item/kg dry mass soil						

* indicated value was extrapolated beyond test concentrations.

Table S12: Effect concentrations (EC_{10}, EC_{50} with 95% confidence interval and NOEC, in mg/kg soil dry mass) for **florfenicol applied via maximum incubation with cattle manure**.

**Species**	***A. cepa***	***A. sativa***	***B. napus***	***S. alba***	***S. lycopersicum***	***P. vulgaris***
Emergence						
NOEC	≥ 50	≥ 50	≥ 50	≥ 50	≥ 50	≥ 50
EC_{10}	n.d.	n.d.	n.d.	n.d.	0.04	n.d.
	(-)	(-)	(-)	(-)	(n.d.)	(-)
EC_{50}	n.d.	n.d.	n.d.	n.d.	n.d.	n.d.
	(-)	(-)	(-)	(-)	(-)	(-)
Post-emergence survival						
NOEC	≥ 50	≥ 50	≥ 50	≥ 50	≥ 50	≥ 50
EC_{10}	n.d.	n.d.	n.d.	n.d.	24.2	n.d.
	(-)	(-)	(-)	(-)	(n.d.)	(-)
EC_{50}	n.d.	n.d.	n.d.	n.d.	> 50	n.d.
	(-)	(-)	(-)	(-)	(n.d.)	(-)
Shoot length						
NOEC	16.7	16.7	16.7	16.7	5.6	≥ 50
EC_{10}	17.6	181	29.3	16.8	16.9	n.d.
	(4.19 - 2731)	(n.d.)	(n.d.)	(13.1 - 20.1)	(n.d.)	(-)
EC_{50}	n.d.	n.d.	n.d.	77.6	n.d.	n.d.
	(-)	(-)	(-)	(67.3 - 95.0)	(-)	(-)
Fresh mass						
NOEC	16.7	≥ 50	16.7	16.7	16.7	5.60
EC_{10}	32.8	61.5	27.0	10.1	14.2	8.14
	(n.d.)	(n.d.)	(n.d.)	(3.31 - 15.8)	(n.d.)	(5.86 - 10.3)
EC_{50}	84.1*	n.d.	68.0*	44.3	36.8	69.7*
	(n.d.)	(-)	(n.d.)	(33.6 - 68.9)	(n.d.)	(58.3 - 88.4)
valid:	yes	yes	yes	no (60% emerged, 100% survived)	no (85% emerged, 82% survived)	yes
application range: 0.62 - 50.0 mg test item/kg dry mass soil						

* indicated value was extrapolated beyond test concentrations.

Table S13: Effect concentrations (EC_{10}, EC_{50} with 95% confidence interval and NOEC, in mg/kg soil dry mass) for **tylosin tartrate applied via freshly spiked pig manure**.

**Species**	***A. cepa***	***A. sativa***	***B. napus***	***S. lycopersicum***	***P. vulgaris***	***T. pratense***
Emergence						
NOEC	≥ 300	≥ 1000	≥ 391	≥ 391	≥ 937	≥ 125
EC_{10}	n.d.	n.d.	66.7	n.d.	n.d.	n.d.
	(-)	(-)	(-)	(-)	(-)	(-)
EC_{50}	n.d.	n.d.	n.d.	n.d.	n.d.	n.d.
	(-)	(-)	(-)	(-)	(-)	(-)
Post-emergence survival						
NOEC	60	700	62.5	156	≥ 937	20
EC_{10}	106	633	40.5	69.9	n.d.	17.9
	(n.d.)	(n.d.)	(n.d.)	(n.d.)	(-)	(n.d.)
EC_{50}	166	n.d.	165	212	n.d.	36.8
	(n.d.)	(n.d.)	(n.d.)	(n.d.)	(-)	(n.d.)
Shoot length						
NOEC	15	400	62.5	62.5	< 40	20
EC_{10}	14.7*	548	109	57	26.3*	15.6
	(0.1 - 40.3)	(532 - 562)	(n.d.)	(0.6 - 105)	(2.2 - 63.8)	(9.3 - 20.4)
EC_{50}	207	1076*	164	171	432	40.7
	(103 - 2205)	(1060 - 1093)	(n.d.)	(79.9 - 395)	(263 - 977)	(34.5 - 49.6)
Fresh mass						
NOEC	30	200	62.5	25	40	10
EC_{10}	18.8	226	101	53	18.1*	8.5
	(0.1 - 38.5)	(188 - 259)	(n.d.)	(49.8 - 56.0)	(1.1 - 43.6)	(7.0 - 9.9)
EC_{50}	62.7	499	131	88.1	142	20.1
	(19.7 - 311)	(464 - 534)	(n.d.)	(84.4 - 92.4)	(72.5 - 253)	(18.4 - 21.9)
valid:	yes	yes	yes	yes	yes	yes
application range:	15 - 300	100 - 1000	15 - 300	15 - 300	40 - 937	5.0 - 391

* indicated value was extrapolated beyond test concentrations.

Table S14: Effect concentrations (EC_{10}, EC_{50} with 95% confidence interval and NOEC, in mg/kg soil dry mass) for **tylosin tartrate applied via freshly spiked cattle manure**.

**Species**	***A. cepa***	***A. sativa***	***B. napus***	***S. lycopersicum***	***P. vulgaris***	***T. pratense***
Emergence						
NOEC	≥ 300	≥ 1000	≥ 391	≥ 391	≥ 937	≥ 125
EC_{10}	n.d.	n.d.	n.d.	n.d.	n.d.	n.d.
	(-)	(-)	(-)	(-)	(-)	(-)
EC_{50}	n.d.	n.d.	n.d.	n.d.	n.d.	n.d.
	(-)	(-)	(-)	(-)	(-)	(-)
Post-emergence survival						
NOEC	60	≥ 1000	156	156	≥ 937	20
EC_{10}	52.4	n.d.	235	164	n.d.	11.6
	(n.d.)	(-)	(131 - 298)	(n.d.)	(-)	(0.1 - 20.4)
EC_{50}	127	n.d.	n.d.	197	n.d.	23.8
	(n.d.)	(-)	(-)	(n.d.)	(-)	(8.8 - 115)
Shoot length						
NOEC	15	200	25	62.5	40	5
EC_{10}	16.2	628	52.8	119	60.5	5.2
	(n.d.)	(101 - n.d.)	(16.2 - 85.4)	(n.d.)	(1.7 - 138)	(4.4 - 6.0)
EC_{50}	113	n.d.	217	161	458	33.4
	(n.d.)	(n.d.)	(158 - 320)	(n.d.)	(252 - 1346)	(30.9 - 36.5)
Fresh mass						
NOEC	15	200	25	62.5	40	< 5.0
EC_{10}	15.7	246	36.7	65.7	25.3*	2.9*
	(n.d.)	(174 - 306)	(25.2 - 44.9)	(65.4 - 66.0)	(1.1 - 59.9)	(1.9 - 3.8)
EC_{50}	46.3	775	78.7	100	177	10.7
	(n.d.)	(697 - 879)	(69.2 - 90.3)	(100 - 101)	(88 - 346)	(9.4 - 12.3)
valid:	yes	yes	yes	yes	yes	yes
application range:	15 - 300	100 - 1000	15 - 300	15 - 300	40 - 937	5.0 - 391

* indicated value was extrapolated beyond test concentrations.

Table S15: Effect concentrations (EC_{10}, EC_{50} with 95% confidence interval and NOEC, in mg/kg soil dry mass) for **tylosin tartrate applied via half-maximum incubation with pig manure.**

**Species**	***A. cepa***	***A. sativa***	***B. napus***	***S. lycopersicum***	***P. vulgaris***	***T. pratense***
Emergence						
NOEC	≥ 300	≥ 1000	≥ 391	≥ 391	≥ 937	≥ 125
EC_{10}	n.d.	874.4	n.d.	n.d.	n.d.	n.d.
	(-)	(n.d.)	(-)	(-)	(-)	(-)
EC_{50}	n.d.	n.d.	n.d.	n.d.	n.d.	n.d.
	(-)	(-)	(-)	(-)	(-)	(-)
Post-emergence survival						
NOEC	≥ 300	≥ 1000	≥ 391	≥ 391	≥ 937	≥ 125
EC_{10}	n.d.	n.d.	n.d.	n.d.	n.d.	n.d.
	(-)	(-)	(-)	(-)	(-)	(-)
EC_{50}	n.d.	n.d.	n.d.	n.d.	n.d.	n.d.
	(-)	(-)	(-)	(-)	(-)	(-)
Shoot length						
NOEC	150	≥ 1000	156	≥ 391	426	20
EC_{10}	n.d.	n.d.	n.d.	n.d.	534	76.6
	(-)	(-)	(-)	(-)	(n.d.)	(-)
EC_{50}	n.d.	n.d.	n.d.	n.d.	n.d.	n.d.
	(-)	(-)	(-)	(-)	(-)	(-)
Fresh mass						
NOEC	≥ 300	≥ 1000	156	≥ 391	426	≥ 125
EC_{10}	92	n.d.	n.d.	n.d.	581	5.5
	(n.d.)	(-)	(-)	(-)	(n.d.)	(0.1 - 18.0)
EC50	n.d.	n.d.	n.d.	n.d.	837	n.d.
	(-)	(-)	(-)	(-)	(n.d.)	(-)
valid:	yes	yes	yes	yes	yes	yes
application range:	15 - 300	100 - 1000	5.0 - 391	5.0 - 391	40 - 937	5.0 - 125

Table S16: Effect concentrations (EC_{10}, EC_{50} with 95% confidence interval and NOEC, in mg/kg soil dry mass) for **tylosin tartrate applied via half-maximum incubation with cattle manure.**

**Species**	***A. cepa***	***A. sativa***	***B. napus***	***S. lycopersicum***	***P. vulgaris***	***T. pratense***
Emergence						
NOEC	≥ 300	≥ 1000	≥ 391	≥ 391	≥ 937	≥ 125
EC_{10}	n.d.	n.d.	n.d.	n.d.	n.d.	n.d.
	(-)	(-)	(-)	(-)	(-)	(-)
EC_{50}	n.d.	n.d.	n.d.	n.d.	n.d.	n.d.
	(-)	(-)	(-)	(-)	(-)	(-)
Post-emergence survival						
NOEC	≥ 300	≥ 1000	≥ 391	≥ 391	≥ 937	≥ 125
EC_{10}	n.d.	n.d.	n.d.	n.d.	n.d.	n.d.
	(-)	(-)	(-)	(-)	(-)	(-)
EC_{50}	n.d.	n.d.	n.d.	n.d.	n.d.	n.d.
	(-)	(-)	(-)	(-)	(-)	(-)
Shoot length						
NOEC	150	≥ 1000	156	156	194	50
EC_{10}	149	n.d.	95.5	79.6	188	49.3
	(108 - 176)	(-)	(n.d.)	(n.d.)	(n.d.)	(n.d.)
EC_{50}	301	n.d.	(-)	(-)	753	n.d.
	(275 - 340)	(-)	n.d.	n.d.	(n.d.)	(-)
Fresh mass						
NOEC	150	≥ 1000	156	156	194	50
EC_{10}	156	n.d.	254	77.5	175	70.6
	(149 - 162)	(-)	(n.d.)	(n.d.)	(n.d.)	(n.d.)
EC_{50}	231	n.d.	326	241	370	123
	(226 - 236)	(-)	(n.d.)	(n.d.)	(n.d.)	(n.d.)
valid:	yes	yes	yes	yes	yes	yes
application range:	15 - 300	100 - 1000	5.0 - 391	5.0 - 391	40 - 937	5.0 - 125

Table S17: Effect concentrations (EC_{10}, EC_{50} with 95% confidence interval and NOEC, in mg/kg soil dry mass) for **tylosin tartrate applied via maximum incubation with pig manure.**

**Species**	***A. cepa***	***A. sativa***	***B. napus***	***S. lycopersicum***	***P. vulgaris***	***T. pratense***
Emergence						
NOEC	≥ 300	≥ 1000	≥ 391	≥ 391	≥ 937	≥ 125
EC_{10}	n.d.	n.d.	n.d.	n.d.	n.d.	n.d.
	(-)	(-)	(-)	(-)	(-)	(-)
EC_{50}	n.d.	n.d.	n.d.	n.d.	n.d.	n.d.
	(-)	(-)	(-)	(-)	(-)	(-)
Post-emergence survival						
NOEC	≥ 300	≥ 1000	≥ 391	≥ 391	≥ 937	≥ 125
EC_{10}	n.d.	n.d.	n.d.	n.d.	n.d.	n.d.
	(-)	(-)	(-)	(-)	(-)	(-)
EC_{50}	n.d.	n.d.	n.d.	n.d.	n.d.	n.d.
	(-)	(-)	(-)	(-)	(-)	(-)
Shoot length						
NOEC	≥ 300	≥ 1000	≥ 391	≥ 391	426	≥ 125
EC_{10}	n.d.	n.d.	n.d.	n.d.	383	n.d.
	(-)	(-)	(-)	(-)	(n.d.)	(-)
EC_{50}	n.d.	n.d.	n.d.	n.d.	n.d.	n.d.
	(-)	(-)	(-)	(-)	(-)	(-)
Fresh mass						
NOEC	≥ 300	≥ 1000	≥ 391	≥ 391	426	≥ 125
EC_{10}	n.d.	n.d.	n.d.	n.d.	553	66.8
	(-)	(-)	(-)	(-)	(n.d.)	(n.d.)
EC_{50}	n.d.	n.d.	n.d.	n.d.	885	n.d.
	(-)	(-)	(-)	(-)	(n.d.)	(n.d.)
valid:	yes	yes	yes	yes	yes	yes
application range:	15 - 300	100 - 1000	5.0 - 391	5.0 - 391	40 - 937	5.0 - 125

Table S18: Effect concentrations (EC_{10}, EC_{50} with 95% confidence interval and NOEC, in mg/kg soil dry mass) for **tylosin tartrate applied via maximum incubation with cattle manure.**

**Species**	***A. cepa***	***A. sativa***	***B. napus***	***S. lycopersicum***	***P. vulgaris***	***T. pratense***
Emergence						
NOEC	≥ 300	≥ 1000	≥ 391	≥ 391	≥ 937	≥ 125
EC_{10}	n.d.	n.d.	n.d.	148	n.d.	n.d.
	(-)	(-)	(-)	(56.3 - 624)	(-)	(-)
EC_{50}	n.d.	n.d.	n.d.	n.d.	n.d.	n.d.
	(-)	(-)	(-)	(-)	(-)	(-)
Post-emergence survival						
NOEC	≥ 300	≥ 1000	≥ 391	≥ 391	≥ 937	≥ 125
EC_{10}	n.d.	n.d.	n.d.	n.d.	n.d.	n.d.
	(-)	(-)	(-)	(-)	(-)	(-)
EC_{50}	n.d.	n.d.	n.d.	n.d.	n.d.	n.d.
	(-)	(-)	(-)	(-)	(-)	(-)
Shoot length						
NOEC	≥ 300	≥ 1000	156	≥ 391	194	≥ 125
EC_{10}	145	n.d.	119	n.d.	365	n.d.
	(n.d.)	(-)	(n.d.)	(-)	(256 - 446)	(-)
EC_{50}	n.d.	n.d.	n.d.	n.d.	n.d.	n.d.
	(-)	(-)	(-)	(-)	(-)	(-)
Fresh mass						
NOEC	≥ 300	≥ 1000	156	156	194	≥ 125
EC_{10}	104	n.d.	78.7	113	202	n.d.
	(n.d.)	(-)	(n.d.)	(n.d.)	(33.6 - 315)	(-)
EC_{50}	n.d.	n.d.	351	n.d.	525	n.d.
	(-)	(-)	(n.d.)	(-)	(352 - 819)	(-)
valid	yes	yes	yes	yes	yes	yes
application range:	15 - 300	100 - 1000	5.0 - 391	5.0 - 391	40 - 937	5.0 - 125

Table S19: Effect concentrations regarding plant fresh mass (EC_{10}, EC_{50} with 95% confidence interval and NOEC, in mg/kg soil dry mass) for **florfenicol and tylosin tartrate**, respectively, applied via half-maximum incubation **under aerobic or anaerobic conditions.**

**Florfenicol**				
**Species**	***Allium cepa***			
**Spiked carrier**	**Pig manure**		**Cattle manure**	
**Incubation condition**	**Manure anaerobic**	**Manure/soil aerobic**	**Manure anaerobic**	**Manure/soil aerobic**
NOEC	5.6	0.6	5.6	<0.60
EC_{10}	5.43	0.54	7.71	0.35
EC_{50}	17.53	1.6	26.45	1.18
**Species**	***Brassica napus***			
**Spiked carrier**	**Pig manure**		**Cattle manure**	
**Incubation condition**	**Manure anaerobic**	**Manure/soil aerobic**	**Manure anaerobic**	**Manure/soil aerobic**
NOEC	16.7	< 0.60	5.6	0.6
EC_{10}	14.08	n.d.	12.05	0.55
EC_{50}	22.5	0.35	20.81	1.1
**Tylosin tartrate**				
**Species**	***Allium cepa***			
**Spiked carrier**	**Pig manure**		**Cattle manure**	
**Incubation condition**	**Manure anaerobic**	**Manure/soil aerobic**	**Manure anaerobic**	**Manure/soil aerobic**
NOEC	≥300	30	150	15
EC_{10}	92	20.3	156	10.7
EC_{50}	n.d.	65.4	156	46
**Species**	***Trifolium pratense***			
**Spiked carrier**	**Pig manure**		**Cattle manure**	
**Incubation condition**	**Manure anaerobic**	**Manure/soil aerobic**	**Manure anaerobic**	**Manure/soil aerobic**
NOEC	≥125	10	50	5
EC_{10}	5.5	7.7	70.6	5.3
EC_{50}	n.d.	29.8	123	16.9
